# Contribution to the African Ladybird Genus *Epipleuria* Fürsch (Coccinellidae: Coccidulini) with Description of a New Genus

**DOI:** 10.3390/insects16050456

**Published:** 2025-04-25

**Authors:** Tomasz Czerwiński, Karol Szawaryn

**Affiliations:** Museum and Institute of Zoology, Polish Academy of Sciences, Twarda 51/55, 00-818 Warsaw, Poland; tczerwinski@miiz.waw.pl

**Keywords:** Coccinelloidea, ladybird beetles, new genus, Afrotropic, taxonomy

## Abstract

The endemic African genus *Epipleuria* Fürsch is re-described, diagnosed and confronted with African species of the closely related genus *Rhyzobius* Stephens. The validity of the genus *Epipleuria* is confirmed. Two new species, *Epipleuria capensis* **sp. nov.** and *Epipleuria tsitsikamma* **sp. nov.**, from the Republic of South Africa are described. Additionally, the male genitalia of *Epipleuria natalensis* Fürsch, 2001, are described and illustrated for the first time. *Epipleuria endroedyi* Fürsch, 2001; *Epipleuria mahnerti* Fürsch, 2001; and *Rhyzobius stillatus* Fürsch, 1992, are transferred to the newly established genus *Pseudoepipleuria* **gen. nov**.

## Simple Summary

The ladybird tribe Coccidulini is one of the most problematic in terms of taxonomy and classification. However, it exhibits a Gondwanan-type distribution, making it one of the most phylogenetically and evolutionarily interesting tribes within the Coccinellidae. One of the neglected genera within this group is the African genus *Epipleuria* Fürsch, which currently comprises 24 species from the Republic of South Africa and Kenya. Since its description, *Epipleuria* has not been properly distinguished from the closely related, globally distributed genus *Rhyzobius* Stephens, which also includes African representatives. The purpose of this study was to redefine *Epipleuria* and investigate whether it represents a distinct, independent lineage or should be synonymized with *Rhyzobius*, as previously suggested in some studies. By analyzing diverse material from several institutions, we examined six species of *Epipleuria*, including two described here as new. Three additional species have been transferred to a newly proposed genus, *Pseudoepipleuria*. Furthermore, the relationships between both genera and *Rhyzobius* are discussed in detail. The results provide new insights that contribute to the ongoing discussion about the relationships among African members of the tribe Coccidulini. This study establishes a new framework for future molecular research.

## 1. Introduction

The tribe Coccidulini Mulsant is a relatively large and widely distributed group of ladybird beetles (Coccinellidae), with its highest diversity in African, Australian and Neotropical regions [1,2]. Despite numerous studies based on both morphological [1,3] and molecular data [4,5,6], the systematic position of the tribe itself and the generic relationships within the tribe are still unresolved, making this group of coccinellid beetles one of the most difficult and problematic within the entire family.

In the most recent phylogeny, based on the most complete molecular dataset to date, Coccidulini are reconstructed as the crown group of Coccinellidae [6]. On the other hand, this tribe has the most diverse fossil record and is the only group of ladybird beetles described from more than one source of fossils. Extinct species of *Rhyzobius* Stephens, 1829, were discovered in Eocene ambers from Oise (~53 Mya) [7] and the Gulf of Gdańsk (~35–48 Mya) [3].

The African realm comprises 41 species of Coccidulini, which are grouped into two genera: *Rhyzobius* Stephens (17 species) and *Epipleuria* Fürsch, 2001 (24 species) [2,8,9,10]. Another endemic African ladybird genus, *Cranophorus* Mulsant, 1850, has traditionally been placed in a separate tribe, Cranophorini Mulsant [11,12]. However, in more recent publications, it is sometimes included in the tribe Coccidulini as well [4,6]. Its phylogenetic relationship with Coccidulini remains unknown.

The genus *Epipleuria* was established by Fürsch [9] with *Epipleuria epipleuralis* (Pope, 1957) as the type species, which was originally placed by Pope [13] in the genus *Rhizobiellus* Oke, 1951. In his paper, Fürsch [9] also included descriptions of 16 newly discovered species. In his subsequent paper [10], he added eight more *Epipleuria* species. Fürsch [9,10] differentiated *Epipleuria* from *Rhyzobius* using several morphological features, such as a wide epipleura, the size of the scutellar shield, the presence of tibial spurs and a dentated tip of the penis. However, after the world revision of the genus *Rhyzobius* by Tomaszewska [2], which appeared to be very morphologically diverse, it remained unclear if *Epipleuria* should be synonymized with the latter or if it is a separate genus. Tomaszewska [2], in her revision of the world species of *Rhyzobius*, re-described all African species but did not provide any information on how to distinguish the two genera. Since Fürsch’s publications, *Epipleuria* has not been the subject of any studies.

In molecular studies, *Epipleuria* was used just once [6], and these results showed that the specimen identified as *Epipleuria* sp. does not form a monophyletic clade with African *Rhyzobius* species but is grouped together with European species of Coccidulini (*Coccidula scutellata* (Herbst), *Rhyzobius litura* (Fabricius)). However, that voucher has not been identified to the species level and is not available for re-examination, so it is not clear if that was a correct generic identification.

Currently, the relationship between the two genera, *Epipleuria* and *Rhyzobius*, remains unclear and unresolved. While the African species of *Rhyzobius* have undergone critical redescriptions, *Epipleuria* still requires revisionary work and comprehensive phylogenetic studies. To partially address this gap, we examined *Epipleuria* specimens from various collections, provided a redescription of the genus and differentiated it from *Rhyzobius*. During this study, we observed that a group of examined species differs from both *Epipleuria* and *Rhyzobius* by a set of characteristics. As a result, we propose establishing a new genus within the African Coccidulini.

## 2. Materials and Methods

This study was based on materials from the following institutions: DNMNH, Ditsong National Museum of Natural History, Pretoria, Republic of South Africa; MIZ, Museum and Institute of Zoology, Warsaw, Poland; MNHW, Museum of Natural History, University of Wrocław, Wrocław, Poland; MZLU, Museum of Zoology, Lund University, Lund, Sweden; NHM, Natural History Museum, London, United Kingdom; and ZSM, Bavarian State Collection of Zoology, Munich, Germany.

The measurements were made using a micrometer attached to a dissecting microscope as follows: (TL) total length, from apical margin of clypeus to apex of elytra; (PL) pronotal length, from the middle of anterior margin to margin of basal foramen; (PW) pronotal width at widest part; (EL) elytral length along suture, including scutellum; (EW) elytral width across both elytra at widest part. Genitalia were dissected, cleared in 10% potassium hydroxide (KOH), examined and photographed in glycerol. After examination, the genitalia were glued on a mounting card and pinned with the respective specimen. Color images were taken using a stereo microscope, Leica MZ 16, with a digital camera IC 3D. The final images were produced using Helicon Focus 5.0 x64 and Adobe^®^ Photoshop CS6 software. Color images of the holotype of *Rhizobiellus epipleuralis* were taken by the staff of the MZLU using the digital cameras Canon EOS R MP-E65 mm f/2.8 1-5x Macro Photo and Canon PowerShot G12 6.1–30.5 mm. MicroCT images of the holotype of *Rhizobiellus epipleuralis* were taken by the staff of the MZLU using the tomograph Nicon XT H 225 with Inspect-X XT 6.11 software. The SEM images were taken in the Laboratory of Scanning Microscopy (MIZ Warsaw) using a scanning electron microscope HITACHI S-3400N under low vacuum conditions. The morphological terminology follows Ślipiński [1] and Lawrence et al. [14].

## 3. Results

Taxonomy

Family Coccinellidae Latreille, 1807.
Subfamily Coccinellinae Latreille, 1807.
Tribe Coccidulini Mulsant, 1846.
Genus *Epipleuria* Fürsch, 2001.
Type species: *Rhizobiellus epipleuralis* Pope, 1957—by original designation.

**Diagnosis.** *Epipleuria* shows close resemblance to other African genera of the tribe Coccidulini, *Rhyzobius* and *Pseudoepipleuria* gen. nov., but it can be distinguished from both via the following characteristics: small or indistinct scutellar shield; wide epipleura (more than 4.0× as wide as corresponding metaepisternum); modified parameres (very narrow, sometimes shortened or reduced); penis broadening toward apex and spermatheca divided into bulbous base and vermiform, curved apex. Additionally, from *Rhyzobius*, it differs by elytra fused along elytral suture (elytra not fused in *Rhyzobius*), lack of hind wings (present in *Rhyzobius*), pronotum with lateral margins bordered (lateral margins without border) and tegminal strut with divided apex (simple in *Rhyzobius*). From *Pseudoepipleuria* gen. nov., its differs by elytra with single sized punctures (double in *Pseudoepipleuria*), epipleura without bordering line (bordering line present along inner margin), tibiae with single spur or without spurs (usually double spurs in *Pseudoepipleuria*), posterior margin of ventrite 5 in males variable in shape, but never rounded (in *Pseudoepipleuria* posterior margin of ventrite 5 rounded), tegmen with penis guide modified, variable in shape (tegmen with penis guide simple, wide at base and tapering toward apex) and penis robust with apex covered with hooks or spines (penis simple, rod-like with apex unmodified in *Pseudoepipleuria*).

**Emended generic description.** TL = 1.3–2.3 mm; EW = 0.9–1.6 mm. Body elongate to broadly oval, moderately to strongly convex. Dorsum covered with dense pubescence and sparse, erect bristles uniformly dispersed through the elytral surface or along lateral margins only. Elytra with single-sized punctures. Body unicolored brown, sometimes with antennae, mouthparts, pronotal margins, legs or abdominal ventrites paler. Wingless.

**Figure 1:**
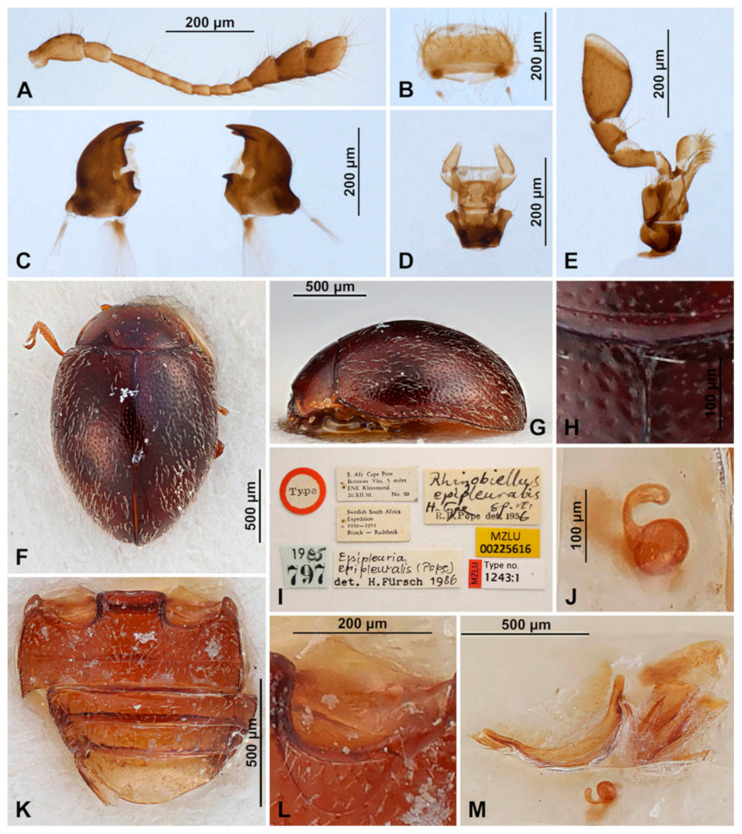
(**A**–**E**) *Epipleuria globosa* Fürsch, head structures. (**A**) Antenna; (**B**) labrum; (**C**) mandibles; (**D**) labium; (**E**) maxilla; (**F**–**M**) *Epipleuria epipleuralis* Pope, female, holotype (MZLU); (**F**) habitus, dorsal; (**G**) habitus, lateral; (**H**) scutellar shield; (**I**) labels; (**J**) spermatheca; (**K**) abdomen; (**L**) abdominal postcoxal line; (**M**) genitalia. Photos: (**F**–**M**) MZLU (modified).

Head partially covered by pronotum; ventral antennal grooves indistinct. Eyes coarsely facetted, dorsally with inner orbits arcuate; interfacetal setae present, sparse and short; eye canthus absent. Antenna (Figure 1A) about 1.0–1.3×, as long as head capsule width, composed of 11 antennomeres; scape swollen; pedicel barrel-shaped, distinctly narrower than scape; antennal club composed of three antennomeres, with two subterminal antennomeres asymmetrical. Anterior clypeal margin straight, without lateral lobes. Labrum entirely exposed, transverse, truncate at apex (Figure 1B). Mandible bidentate at apex, prostheca well developed, setose; molar teeth asymmetrical (Figure 1C). Maxilla with cardo transverse (Figure 1E), usually reaching outside of mouth cavity; maxillary stipes deeply foveate to accommodate maxillary palp in repose, basistipes with lateral carina distinctly sinuate; lacinia elongate, covered with moderately long setae; lacinia narrow, galea C-shaped, densely setose apically; maxillary palp composed of four palpomeres, palpomere 1 small, palpomere 2 distinctly longer than palpomere 3; terminal palpomere subparallel, weakly expanded apically. Submentum distinct, subrectangular, its anterior margin straight. Labium (Figure 1D) with mentum transverse, anterior edge truncate or emargined; ventral surface with horseshoe-shaped median impression; prementum subquadrate or weakly elongate; labial palps separated by a distance of about 1.0–1.5× width of palpiger; palp composed of three palpomeres, terminal palpomere as long and as broad as penultimate, apically pointed.

Prothorax. Pronotum with anterior corners obtuse, not swollen with regular margin anterior margin with fine bordering line; lateral and posterior margins entirely bordered.

Prothoracic hypomeron sparsely hairy, smooth, sometimes with short groove near its anterior margin. Prosternum in front of coxa about 0.6–0.7× as long as coxal longitudinal diameter at the same position; anterior pronotal margin usually with bordering line incomplete medially; notosternal suture distinct or indistinct; prosternal process 0.4–0.6× as wide as corresponding procoxal width, with truncate apex, its surface with carinae present; procoxal cavity transverse, without lateral slit, with or without bordering line.

Pterothorax. Mesoventrite transverse, its anterior edge emarginate medially with complete raised border. Meso-metaventral articulation with suture visible; its junction straight or arcuate anteriorly. Scutellar shield very small, invisible in some species. Both elytra fused along elytral suture. Elytra with sides rounded; lateral margins narrow; humeral calli indistinct; elytral epipleuron in basal half more than 4.0× as wide as corresponding metaepisternum, obsolete in apical half, not foveate, inner margin without bordering line. Metaventrite with discrimen incomplete; metaventral postcoxal lines joined at middle forming straight or arcuate line, straight or slightly recurved laterally, complete. Metendosternite reduced, stalk very short, with longitudinal flange not divided (without cross), anterior tendons widely separated.

Legs. Trochanters simple or angulately produced; tibiae simple; in males, hind tibiae sometimes broadened; tibial apices of mid and hind legs with single spur in both sexes or sometimes without spurs. Tarsi pseudotrimerous; in males, fore and mid-tarsal claws bifid, hind tarsal claws with subquadrate basal tooth; in females, all tarsal claws with subquadrate basal tooth.

Abdomen. Abdomen with five or six ventrites; abdominal postcoxal lines separate medially, complete, rounded; ventrite 1 below coxae longer than ventrite 2. In males, posterior margin of ventrite 5 variable in shape, but never rounded. Males in some species with short spines on anterolateral margins of ventrites 3–5 (absent in females). Tergite VIII rounded apically; sternite VIII truncate or emargined apically. Ventrite 5 in females with posterior margin arcuate; tergite VIII and sternite VIII rounded apically.

Male terminalia and genitalia. Sternite IX with apodeme narrow, rod-like, base of apodeme simple or widened; tergite X short, transverse. Tegmen usually symmetrical, shape of penis guide variable. Parameres very narrow, simple, usually as long as penis guide, in some species shortened or reduced; tegminal strut broad, robust, its apex divided, v-shaped. Penis capsule variable; penis always broadening toward apex; apex usually with hooks or spines.

Female genitalia. Proctiger elongate with hind margin rounded, covered with long hairs. Coxites subtriangular in shape, long and narrow; apices covered with sparse setae; styli distinct, bearing long setae. Sperm duct uniform in diameter, infundibulum absent; spermatheca with bulbous base and vermiform, curved apex, without clear nodulus and ramus, spermathecal accessory gland reduced.

**Distribution.** Republic of South Africa, Lesotho.

Current composition of the genus (24 species).

[Species examined in this study in bold, *—indicates the type species.]

***Epipleuria capensis* sp. nov.;** *Epipleuria caputabdita* Fürsch, 2007; *Epipleuria epipleuralis* (Pope, 1957)*; *Epipleuria globosa* Fürsch, 2001; *Epipleuria gussmannae* Fürsch, 2001; *Epipleuria hirsutula* Fürsch, 2007; *Epipleuria hirta* Fürsch, 2007; ***Epipleuria inexspectata* Fürsch, 2001;** *Epipleuria katbergensis* Fürsch, 2001; *Epipleuria lapidaria* Fürsch, 2007; *Epipleuria longissima* Fürsch, 2001; *Epipleuria namaquaensis* Fürsch, 2001; ***Epipleuria natalensis* Fürsch, 2001;** *Epipleuria parcepunctata* Fürsch, 2001; *Epipleuria parva* Fürsch, 2001; *Epipleuria popei* Fürsch, 2001; *Epipleuria punctillum* Fürsch, 2001; *Epipleuria rufosuturalis* Fürsch, 2001; *Epipleuria rugata* Fürsch, 2007; *Epipleuria ruthmuellerae* Fürsch, 2007; *Epipleuria saxicola* Fürsch, 2007; *Epipleuria trianguliloba* Fürsch, 2001; ***Epipleuria tsitsikamma* sp. nov.;** *Epipleuria ventricosa* Fürsch, 2001.

**Figure 2:**
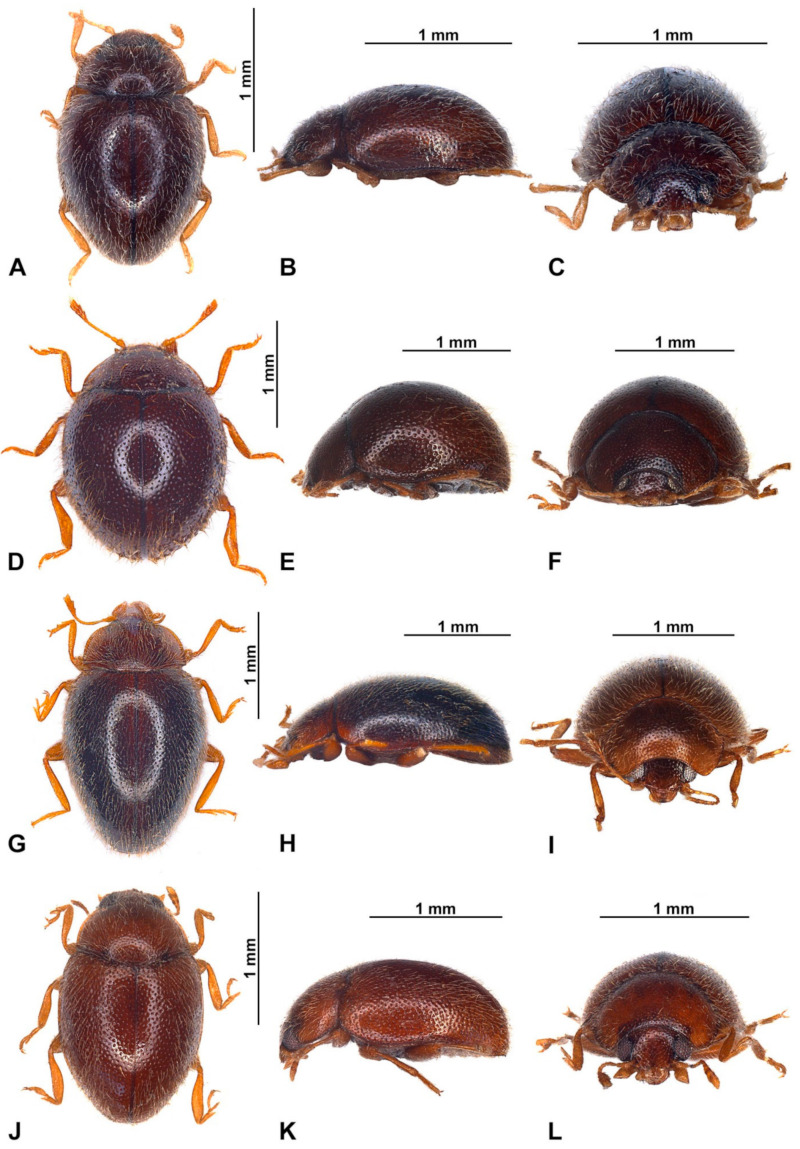
Habitus of *Epipleuria* Fürsch species. (**A**–**C**) *Epipleuria capensis* sp. nov., holotype (ZSM). (**A**) Dorsal; (**B**) lateral; (**C**) frontal; (**D**–**F**) *Epipleuria globosa* Fürsch (MNHW); (**D**) dorsal; (**E**) lateral; (**F**) frontal; (**G**–**I**) *Epipleuria inexpectata* Fürsch (MNHW); (**G**) dorsal; (**H**) lateral; (**I**) frontal; (**J**–**L**) *Epipleuria natalensis* Fürsch (NHM); (**J**) dorsal; (**K**) lateral; (**L**) frontal.

**Figure 3:**
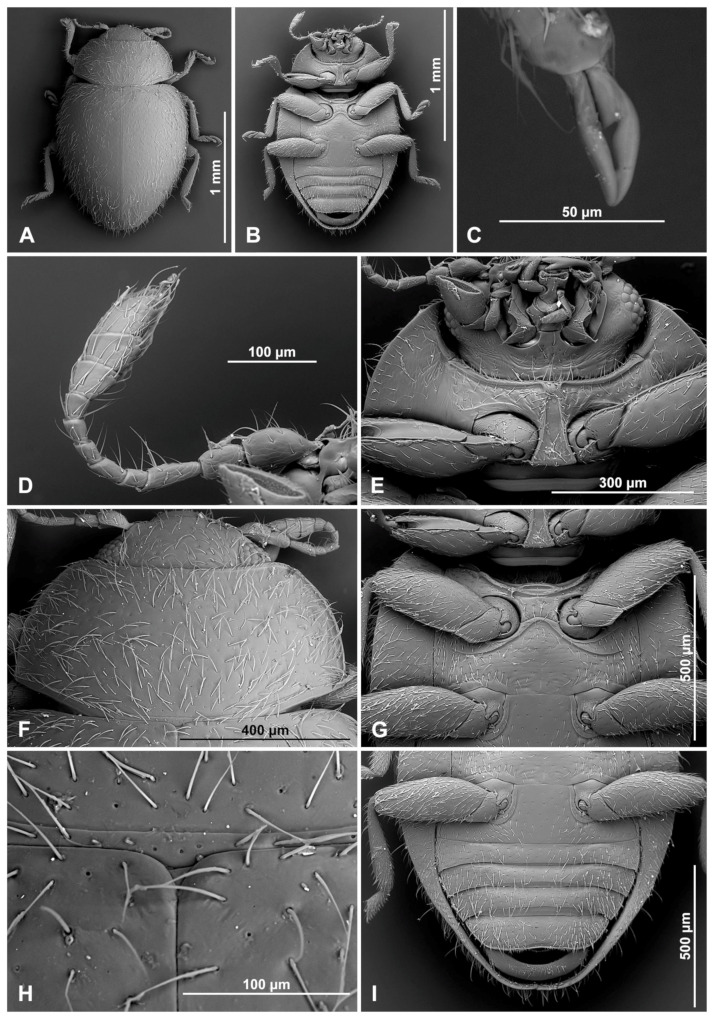
*Epipleuria capensis* sp. nov., male, holotype (ZSM). (**A**) Dorsal. (**B**) ventral. (**C**) hind tarsal claws. (**D**) antenna. (**E**) head and prothorax, ventral. (**F**) pronotum. (**G**) meso- and metathorax, ventral. (**H**) scutellar shield. (**I**) abdomen.

**Figure 4:**
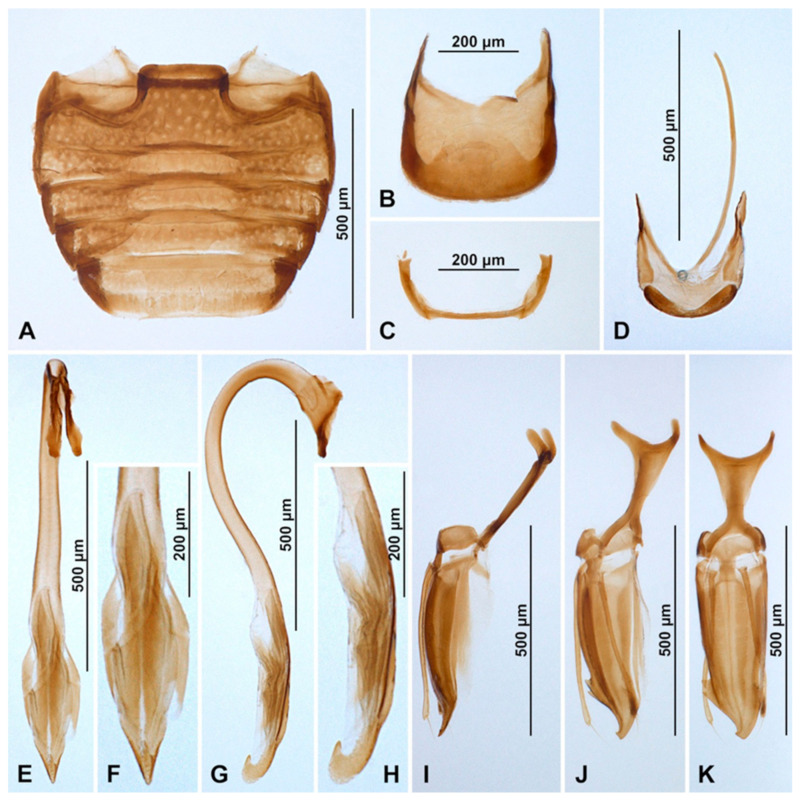
*Epipleuria capensis* sp. nov., male, holotype (ZSM). (**A**) abdomen. (**B**) tergite VIII. (**C**) sternite VIII. (**D**) segment IX. (**E**) penis, inner. (**F**) penis tip, inner. (**G**) penis, lateral. (**H**) penis tip, lateral. (**I**) tegmen, lateral. (**J**) tegmen, details. (**K**) tegmen, inner.

***Epipleuria capensis* sp. nov.** (Figure 2A–C, Figure 3A–I and Figure 4A–K).
ZooBank. urn:lsid:zoobank.org:act:AD0F5F48-3361-47B5-96B4-6647DAFB8927

**Etymology.** The specific epithet refers to the Western Cape, a South African province where the holotype was collected.

**Material examined.** HOLOTYPE: male “S.Afr., Cape Ruitersbos 33.53S - 22.01E/ 20.11.1978; WB: 52 grassnetting leg. W. Breytenbach/ Coll. Fürsch ZSM 2016/ SNSB-Zoologische Staatssammlung München Coleoptera ID # ZSM-COL-00592” (ZSM).

**Diagnosis.** This is very distinctive species, easily distinguished from other *Epipleuria* species by having abdominal postcoxal lines very close to coxal cavities; metaventral postcoxal lines joined at the middle, forming an arcuate line; and an asymmetric shape of the penis guide.

**Description.** TL = 1.6 mm; EW = 1.1 mm; TL/EW = 1.4; PL/PW = 0.5; EL/EW = 1.1. Body elongate oval (Figure 2A), moderately convex (Figure 2B). Dorsum covered with dense, pale pubescence and sparse, erect bristles along lateral margin (Figure 3A). Body unicolored dark brown, with only antennae, mouthparts, legs, and last abdominal ventrite paler, yellowish brown.

Head. Interocular distance about 0.7× head width across eyes (Figure 2C). Antenna (Figure 3D) about 1.3× as long as head capsule width; scape swollen, about 1.2 times longer than pedicel; pedicel barrel-shaped; antennomere 3 elongate, about 2.5 times longer than antennomere 4; antennomere 4 subquadrate and about 0.7× as long as antennomere 5; antennomeres 6 and 7 about 1.2× as long as wide; antennomere 8 subquadrate; antennal club composed of three antennomeres, with two subterminal antennomeres asymmetrical, terminal antennomere subquadrate, about 1.4× as long as penultimate antennomere, its apex truncate. Maxilla with cardo transverse, reaching outside of mouth cavity. Prementum weakly elongate; labial palps separated by a distance of about equal to width of palpiger.

Prothorax. Anterior margin of pronotum with fine bordering line; lateral and posterior margins entirely bordered (Figure 3F). Prothoracic hypomeron sparsely hairy, smooth, with short groove along its anterior margin (Figure 3E). Prosternum in front of coxa about 0.6× as long as coxal longitudinal diameter at the same position; anterior pronotal margin not produced medially, with bordering line incomplete medially; notosternal suture indistinct; prosternal process narrow, about 0.5× as wide as corresponding procoxal width, with truncate apex, its surface with carinae subparallel, joined roundly just before prosternal margin; procoxal cavity transverse, with bordering line incomplete, broadly separated from coxal cavity.

Pterothorax. Mesoventral process in middle about 0.9× as wide as corresponding coxal diameter; meso-metaventral articulation with suture visible; its junction arcuate anteriorly (Figure 3G). Scutellar shield very small but visible, triangular (Figure 3H). Elytra with sides rounded; lateral margins narrow, visible from above only in basal half; elytral epipleuron in basal half about 4.0× as wide as corresponding metaepisternum (Figure 3B). Metaventrite with discrimen incomplete; metaventral postcoxal lines joined at middle forming arcuate line, straight laterally, complete (Figure 3G).

Legs. Trochanters somewhat angulately produced; tibiae simple; tibial apices of mid and hind legs with single spur. Fore and mid-tarsal claws bifid, hind tarsal claws with subquadrate basal tooth (Figure 3C).

Abdomen with five ventrites (Figure 3I and Figure 4A); abdominal postcoxal lines separate medially, complete, rounded, very close to coxal cavity; ventrite 1 below coxae about 1.4 times longer than ventrite 2; ventrite 4 about 1.4 times longer than ventrite 3; ventrites 4 and 5 subequal in length. Posterior margin of ventrite 5 shallowly emargined. Spines on margins of ventrites 3–5 absent. Tergite VIII with wide, semicircular lobe in anterior part and rounded apically (Figure 4B); sternite VIII narrow, truncate apically (Figure 4C).

Male terminalia and genitalia. Sternite IX (Figure 4D) with apodeme narrow, rod-like, base of apodeme simple, partially membranous; tergite X short, transverse. Tegmen in inner view (Figure 4K) with penis guide wide, asymmetrical, with cornuate lobe on lateral margin (Figure 4J); lateral sides of penis guide strongly upturned inwards; penis guide in lateral view (Figure 4I) wide, partially membranous, its tip slightly recurved and pointed. Parameres very narrow, simple, about 0.9× as long as penis guide, its apices covered with long setae; tegminal strut broad, robust, its base and apex divided, v-shaped. Penis capsule (Figure 4G) with only inner arm well developed, outer arm reduced; penis broadening toward apex (Figure 4E); apex flattened, partially membranous, covered with very tiny spines, asymmetrical in inner view (Figure 4F), tip in lateral view strongly curved (Figure 4H).

Female not studied.

**Distribution.** Republic of South Africa: Western Cape.

**Remarks.** The holotype specimen has been examined by Fürsch [9] and initially identified as *Epipleuria epipleuralis* (Pope). However, after a detailed re-examination of its external and genital features and a comparison with the holotype, Fürsch’s original drawings and redescription of *E*. *epipleuralis*, it became evident that it does not belong to this species. We found only a single male specimen which is described here as a new species.

**Figure 5:**
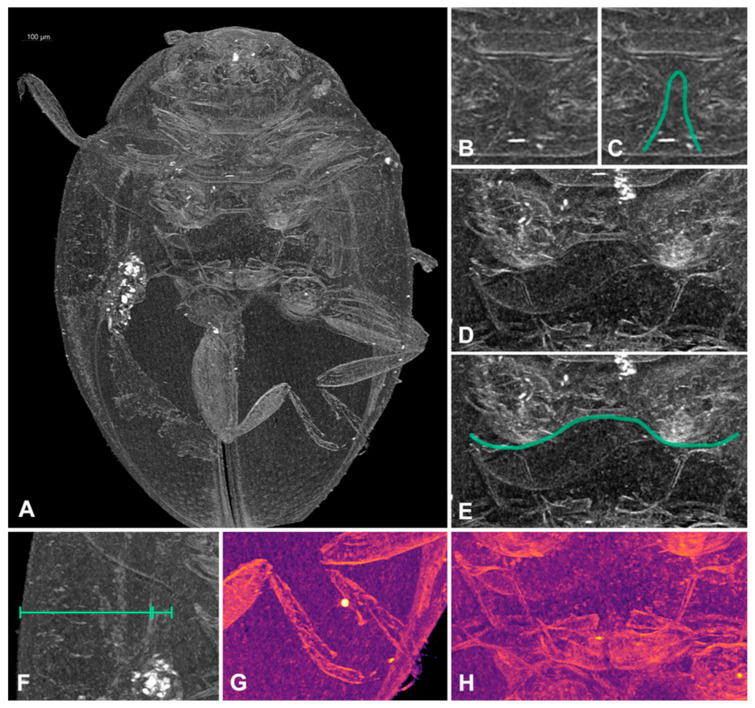
*Epipleuria epipleuralis* (Pope), MicroCT images, female, holotype (MZLU). (**A**) habitus, ventral. (**B**) prosternal process. (**C**) prosternal process lines, outline. (**D**) meso- and metathorax, ventral. (**E**) metathoracic prosternal lines, outline. (**F**) width ratio of epipleura and metaepisternum. (**G**) hind tarsi. (**H**) metendosternite.

***Epipleuria epipleuralis* (Pope, 1957)** (Figure 1F–M and Figure 5A–H).
*Rhizobiellus epipleuralis* Pope, 1957: 304.
*Epipleuria epipleuralis*: Fürsch 2001: 11.

**Material examined.** HOLOTYPE (Figure 1I): female “Type [red disc]/ S Afr. Cape Prov. Botrivier Vlei, 5 miles ENE Kleinmond, 20.XI.50. No. 90/ Swedish South Africa Expedition 1950–1951 Brink–Rudebeck/ Rhizobiellus epipleuralis Holotype sp.n. R.D.Pope det. 1956/ 1985 797 [blue rectangle]/ Epipleuria epipleuralis (Pope) det. H. Fürsch 1986/ MZLU 00225616 [yellow rectangle]/ MZLU Type no. 1243:1” (MZLU).

**Redescription.** TL = 1.8–2.0 mm; EW = 1.2–1.3 mm; TL/EW = 1.4; PL/PW = 0.40; EL/EW = 1.2. Body broadly oval (Figure 1F), convex (Figure 1G). Dorsum covered with dense, erect, pale pubescence. Punctures on pronotum more dense than on elytra. Body unicolored dark brown, with antennae, mouthparts and legs, yellowish brown.

Head partially covered by pronotum. Interocular distance about 0.7× head width across eyes. Eyes coarsely facetted. Anterior clypeal margin straight, without lateral lobes.

Prothorax. Anterior margin of pronotum with fine bordering line; lateral and posterior margins with entire border. Prothoracic hypomeron smooth (Figure 5A). Prosternal process narrow, about 0.5× as wide as corresponding procoxal width, with truncate apex, its surface with carinae subparallel, joined roundly just before prosternal margin (Figure 5B,C).

Pterothorax. Mesoventral process in middle about equal as corresponding coxal diameter; meso-metaventral articulation with suture visible; its junction straight. Scutellar shield small, triangular (Figure 1H). Elytra with sides rounded; lateral margins narrow, visible from above only in basal half; elytral epipleuron in basal half about 6.0× as wide as corresponding metaepisternum (Figure 5F), obsolete in apical half, not foveate. Metaventrite with discrimen incomplete (Figure 5H); metaventral postcoxal lines joined at middle forming straight line, complete and recurved (Figure 5D,E).

Legs. Trochanters somewhat angulately produced; tibiae simple. Tarsi pseudotrimerous (Figure 5G), tarsal claws with subquadrate basal tooth.

Abdomen (Figure 1K). Abdomen with five ventrites; abdominal postcoxal lines (Figure 1L) separate medially, complete, sinuate laterally, posteriorly reaching mid-length of ventrite 1; ventrite 1 below coxae about equal as long as ventrite 2; ventrites 3 and 4 subequal in length; Ventrite 5 slightly longer than ventrite 4, its posterior margin strongly arcuate; tergite VIII and sternite VIII rounded apically.

Female genitalia (Figure 1M). Proctiger elongate with hind edge rounded. Coxites subtriangular, long and narrow. Sperm duct uniform in diameter; infundibulum absent; spermatheca (Figure 1J) with bulbous base and vermiform, curved apex, without clear nodulus and ramus, spermathecal accessory gland indistinct.

Male not studied.

**Distribution.** Republic of South Africa: Western Cape.

**Remarks.** In his 2001 paper [9], Fürsch studied a range of specimens from various localities, which he assigned to *E*. *epipleuralis* (Pope). A few of these specimens were subsequently deposited in the ZSM collection in Munich. Fürsch drew the male and female genitalia of *E*. *epipleuralis* based on specimens from the Golden Gate Highlands N.P. However, the holotype comes from Bot River town near Cape Town, which is over 1000 km apart.

We examined three specimens from the Golden Gate Highlands N.P. from the Fürsch collection and compared them with the microCT scans and color pictures of the holotype of *Rhizobiellus epipleuralis* (MZLU). We noticed several differences, including paler coloration, differences in body shape, indistinct scutellar shield and different shape of abdominal postcoxal lines. Considering that *Epipleuria* specimens from various localities (even relatively close ones) often represent different species, we assume that the specimens from the Golden Gate Highlands N.P. represent a different, undescribed species. However, among the examined specimens, we found two females and a single male without an abdomen. Thus, we decided not to describe these specimens as we were unable to examine the genitalia in detail. As a result, the drawings of male genitalia in Fürsch’s 2001 paper do not represent *E. epipleuralis*.

Moreover, other specimens from the Fürsch collection belong to different species as well. The specimen from Ruitersbos is described here as *Epipleuria capensis* sp. nov. Another specimen from Elandsbay is a female of an unknown species, and the specimen from Vogelklip is missing genitalia. To conclude, *E. epipleuralis* is only known from the holotype so far, which is a female. There is a need to search for additional specimens from the type locality to describe a male for this species.

**Figure 6:**
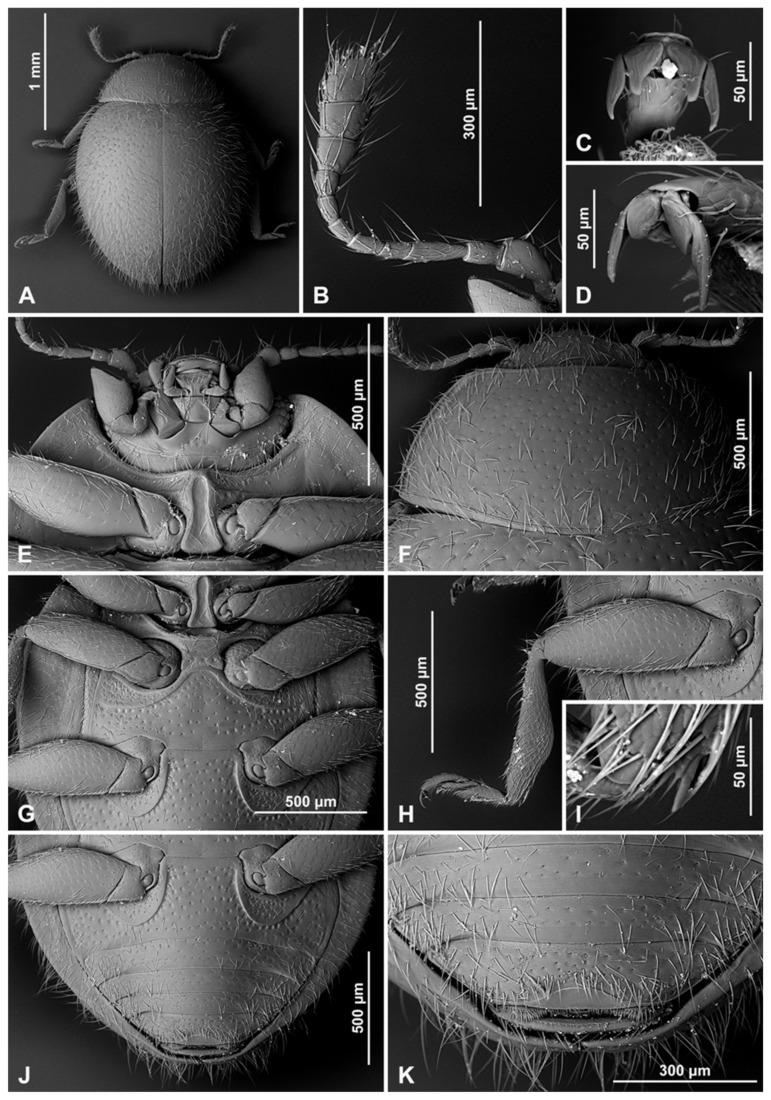
*Epipleuria globosa* Fürsch, male (MNHW). (**A**) Dorsal; (**B**) antenna; (**C**) fore tarsal claws; (**D**) hind tarsal claws; (**E**) head and prothorax, ventral; (**F**) pronotum; (**G**) meso- and metathorax, ventral; (**H**) hind leg; (**I**) tibial spur on hind leg; (**J**) abdomen; (**K**) distal part of abdomen.

**Figure 7:**
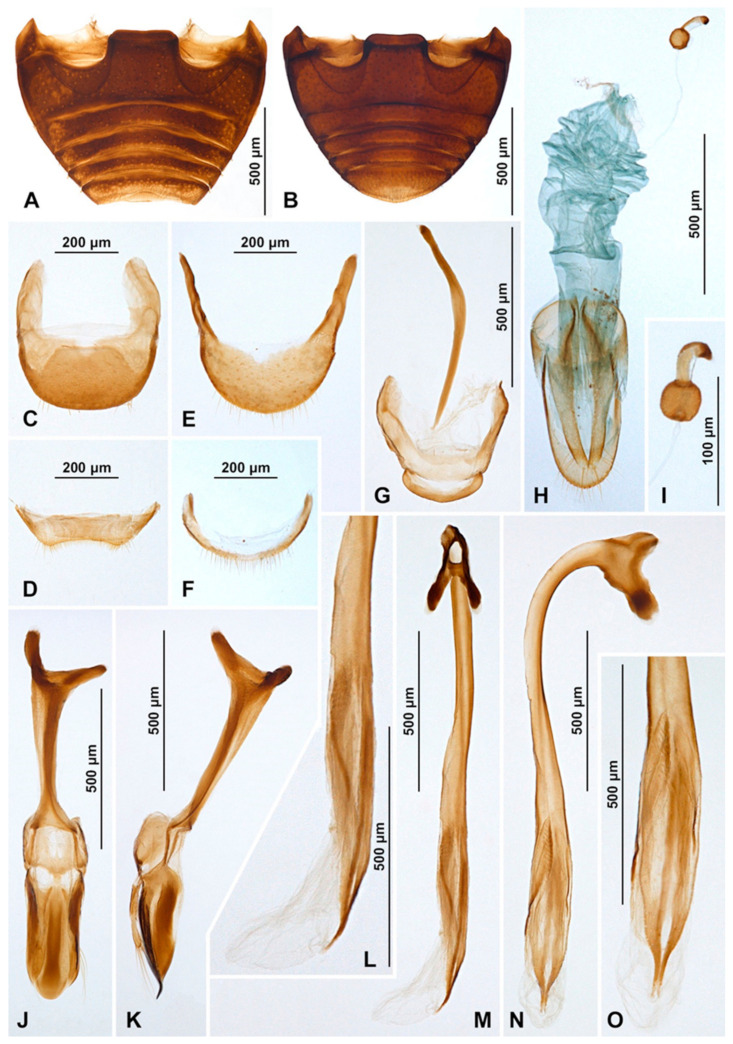
*Epipleuria globosa* Fürsch (MNHW). (**A**) Male abdomen; (**B**) female abdomen; (**C**) male tergite VIII; (**D**) male sternite VIII; (**E**) female tergite VIII; (**F**) female sternite VIII; (**G**) male segment IX; (**H**) female genitalia; (**I**) spermatheca; (**J**) tegmen, inner; (**K**) tegmen, lateral; (**L**) penis tip, inner; (**M**) penis, inner; (**N**) penis, lateral; (**O**) penis tip, inner.

***Epipleuria globosa* Fürsch, 2001** (Figure 2D–F, Figure 6A–K and Figure 7A–O).
*Epipleuria globosa* Fürsch, 2001: 13.

**Material examined.** “RSA (E) E Cape 36 m -31.6511/29.5086 Silaka Reserve 28.11.2019 night collecting leg. M. Wanat” (two males; MNHW); same data collection as preceding, but 27.11.2019 (two males, three females; MNHW; one male, two females; MIZ).

**Redescription.** TL = 1.8–2.0 mm; EW = 1.4–1.6 mm; TL/EW = 1.3; PL/PW = 0.40; EL/EW = 1.0. Body broadly oval (Figure 2D and Figure 6A), strongly convex, hemispherical (Figure 2E). Dorsum covered with dense, erect, yellowish pubescence. Body unicolored dark brown, with only antennae, mouthparts, legs and epipleura paler, yellowish brown.

Head. Interocular distance about 0.6× head width across eyes (Figure 2F). Antenna (Figure 6B) about 1.3× as long as head capsule width; scape swollen, about 2 times longer than pedicel; pedicel barrel-shaped; antennomere 3 elongate, about 2 times longer than antennomere 4; antennomere 4 about 1.6× as long as wide and about 0.8× as long as antennomere 5; antennomeres 6 and 7 about 1.6× as long as wide; antennomere 8 subquadrate; antennal club composed of three antennomeres, with two subterminal antennomeres asymmetrical, terminal antennomere subquadrate, about 1.4× as long as penultimate antennomere, its apex truncate. Maxilla with cardo subquadrate, slightly reaching outside of mouth cavity. Prementum subquadrate; labial palps separated by a distance of about equal to width of palpiger.

Prothorax. Anterior margin of pronotum with fine bordering line; lateral and posterior margins with entire border (Figure 6F). Prothoracic hypomeron sparsely hairy, smooth, without groove or concavity (Figure 6E). Prosternum in front of coxa about 0.7× as long as coxal longitudinal diameter at same position; anterior pronotal margin distinctly produced medially, with visible bordering line; notosternal suture visible, straight; prosternal process narrow, about 0.5× as wide as corresponding procoxal width, with truncate apex, its surface with carinae subparallel, joined roundly just before prosternal margin; procoxal cavity transverse, with bordering line complete, broadly separated from coxal cavity.

Pterothorax. Mesoventral process in middle about 0.8× as wide as corresponding coxal diameter; meso-metaventral articulation with suture visible; its junction arcuate anteriorly (Figure 6G). Scutellar shield small, triangular, slightly elongate, its surface smooth (Figure 6F). Elytra with sides rounded; lateral margins narrow, not visible from above (Figure 6A); elytral epipleuron in basal half about 5.0× as wide as corresponding metaepisternum, obsolete in apical half. Metaventrite with discrimen incomplete; metaventral postcoxal lines joined at middle forming straight line, straight laterally, complete.

Legs. Trochanters somewhat angulately produced; in males, hind tibiae broadened (Figure 6H); in females, simple. Tibial apices of mid and hind legs with single spur in both sexes (Figure 6I). Tarsi pseudotrimerous, in male fore and mid-tarsal claws bifid (Figure 6C), hind tarsal claws with subquadrate basal tooth (Figure 6D); in female claws in all legs with subquadrate basal tooth.

Abdomen. Abdomen with five ventrites in both sexes (Figure 6J and Figure 7A,B); abdominal postcoxal lines separate medially, complete, rounded, deep, posteriorly reaching more than mid-length of ventrite 1; ventrite 1 below coxae about 1.5 times longer than ventrite 2; ventrites 3 and 4 subequal in length; ventrite 5 in male slightly longer than ventrite 4, its posterior margin truncate and surface with shallow postero-median concavity covered with admedian setae (Figure 6K and Figure 7A); tergite VIII rounded (Figure 7C) and sternite VIII shallowly emargined apically (Figure 7D). Anterolateral margins of ventrites 3–5 with short spines. Ventrite 5 in female distinctly longer than ventrite 4, its posterior margin arcuate (Figure 7B); tergite VIII (Figure 7E) and sternite VIII (Figure 7F) rounded apically.

Male terminalia and genitalia. Sternite IX (Figure 7G) with apodeme robust, rod-like, without additional sclerites at base of apodeme; tergite X short, transverse. Tegmen in inner view (Figure 7J) with penis guide symmetrical, lateral sides of penis guide strongly upturned inwards, tip broadly rounded; penis guide in lateral view (Figure 7K) wide, tapering into tip, tip recurved and pointed. Parameres developed, simple, about 0.6× as long as penis guide, its apices covered with long setae; tegminal strut broad, robust, its base broadened and apex divided, v-shaped. Penis capsule with outer and inner arms well developed (Figure 7N); penis broadening toward apex (Figure 7M,N); apex partially membranous, covered with very tiny denticles, asymmetrical in inner view (Figure 7L) and symmetrical in lateral view (Figure 7O), its tip pointed and divided.

Female genitalia (Figure 7H). Proctiger elongate with hind edge rounded, covered with long hairs. Coxites subtriangular in shape, long and narrow; apical margins with row of sparse setae; styli distinct, bearing long setae. Sperm duct uniform in diameter, very long; infundibulum absent; spermatheca (Figure 7I) with bulbous base and vermiform, curved apex, without clear nodulus and ramus, spermathecal accessory gland reduced.

**Distribution.** Republic of South Africa: Eastern Cape.

**Figure 8:**
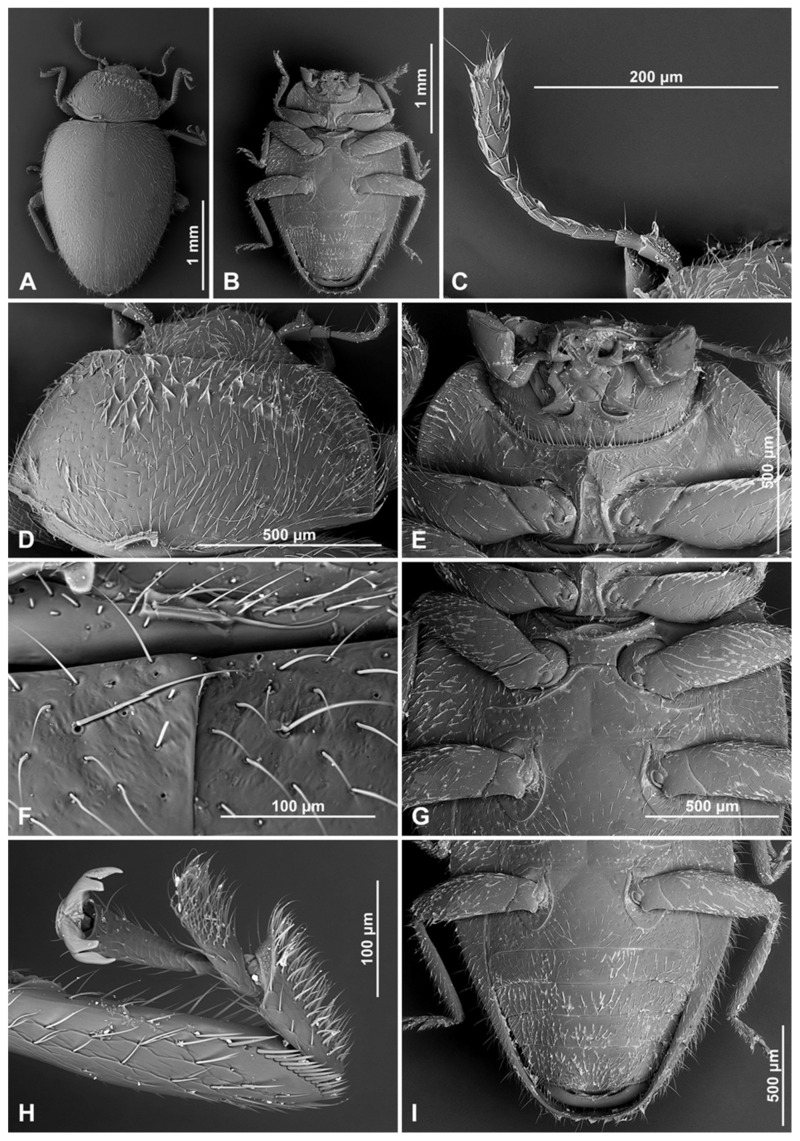
*Epipleuria inexpectata* Fürsch, male (MNHW). (**A**) Dorsal; (**B**) ventral; (**C**) antenna; (**D**) pronotum; (**E**) head and prothorax, ventral; (**F**) scutellar shield; (**G**) meso- and metathorax, ventral; (**H**) mid tarsus; (**I**) abdomen.

**Figure 9:**
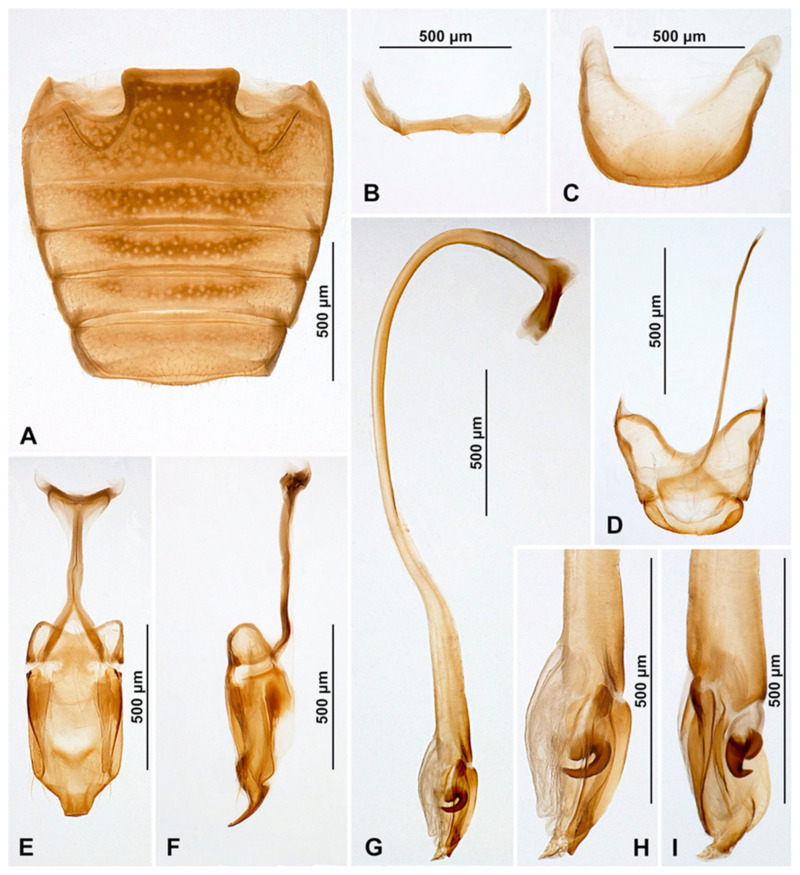
*Epipleuria inexpectata* Fürsch, male (MNHW). (**A**) Abdomen; (**B**) sternite VIII; (**C**) tergite VIII; (**D**) segment IX; (**E**) tegmen, inner; (**F**) tegmen, lateral; (**G**) penis, lateral; (**H**) penis tip, lateral; (**I**) penis tip, inner.

***Epipleuria inexpectata* Fürsch, 2001** (Figure 2G–I, Figure 8A–I and Figure 9A–I).
*Epipleuria inexpectata* Fürsch, 2001: 16.

**Material examined.** HOLOTYPE: male “Mossel May, Cape Province. June, 1921./ S. Africa. R.E. Turner. Brit. Mus. 1921–294./ Holotype Epipleuria inexpectata Fürsch 1986” (NHM). Other material: “RSA/ 32 W Cape 530 m -33.7127 S/22.2981 E Klein Karoo: N12 Rd 15 km S Oudtshoorn roadside karoo vegetation 30.11.2013 leg. R. Ruta” (two males; MNHW; one male MIZ).

**Description.** TL = 2.0–2.1 mm; EW = 1.3–1.4 mm; TL/EW = 1.5; PL/PW = 0.5; EL/EW = 1.2. Body elongate ovate (Figure 2G and Figure 8A), moderately convex (Figure 2H). Dorsum covered with long, pale pubescence and sparse, erect bristles along lateral margin. Body with ventral surface predominantly dark brown, ventral surface, antennae, mouthparts, legs, pronotum along margins and elytra along margins and suture paler, yellowish brown.

Head. Interocular distance about 0.6× head width across eyes (Figure 2I). Antenna (Figure 8C) about 1.1× as long as head capsule width; scape swollen, about 1.6 times longer than pedicel; antennomere 3 elongate, about 1.8 times longer than antennomere 4; antennomere 4 as long as antennomere 5; antennomere 6 about 1.2× as long as wide and antennomere 7 about 1.8× as long as wide; antennomere 8 subquadrate; antennal club elongate, composed of three antennomeres, with two subterminal antennomeres asymmetrical, terminal antennomere elongate, about 1.7× as long as penultimate antennomere, its apex truncate. Maxilla with cardo transverse, distinctly reaching outside of mouth cavity. Labium with mentum transverse, anterior edge emargined; labial palps separated by a distance of about 1.5× width of palpiger.

Prothorax. Anterior margin of pronotum with fine bordering line; lateral and posterior margins with entire border (Figure 8D). Prothoracic hypomeron sparsely hairy, smooth (Figure 8E). Prosternum in front of coxa about 0.6× as long as coxal longitudinal diameter at the same position; anterior pronotal margin not produced medially, with bordering line incomplete medially; notosternal suture distinct; prosternal process narrow, about 0.4× as wide as corresponding procoxal width, with truncate apex, its surface with carinae converging, Y-shaped; procoxal cavity transverse, with bordering line incomplete, broadly separated from coxal cavity.

Pterothorax. Mesoventral process in middle about equal to corresponding coxal diameter; meso-metaventral articulation with suture visible; its junction straight (Figure 8G). Scutellar shield indistinct (Figure 8F). Elytra with sides rounded, its apex elongate; lateral margins narrow, visible from above only in basal half; elytral epipleuron in basal half about 5.0× as wide as corresponding metaepisternum, obsolete in apical half, not foveate (Figure 8B). Metaventrite with discrimen incomplete; metaventral postcoxal lines joined at middle forming straight line, straight laterally, complete (Figure 8G).

Legs. Trochanters somewhat angulately produced; tibiae simple; tibial apices of mid and hind legs without spurs (Figure 8H). Fore and mid-tarsal claws bifid, hind tarsal claws with subquadrate basal tooth.

Abdomen with five ventrites (Figure 8I and Figure 9A); abdominal postcoxal lines separate medially, complete, rounded, posteriorly reaching about 0.5 times of length of ventrite 1; ventrite 1 below coxae about 1.4 times longer than ventrite 2; ventrites 2–4 equal in length; ventrite 5 about 1.4 times longer than ventrite 4. Posterior margin of ventrite 5 truncate, distinctly bordered, slightly rounded at the middle (Figure 9A). Spines on margins of ventrites 3–5 absent. Tergite VIII truncate apically (Figure 9C); sternite VIII narrow, truncate apically (Figure 9B).

Male terminalia and genitalia. Sternite IX (Figure 9D) with apodeme narrow, rod-like, base of apodeme widened, partially membranous; tergite X transverse. Tegmen in inner view (Figure 9E) with penis guide wide, symmetrical, its lateral margins parallel and tip truncate; penis guide in lateral view (Figure 9F) wide, partially membranous, its inner margin sinuate, tip recurved and pointed. Parameres converging, about 0.7× as long as penis guide, its apices covered with long setae; tegminal strut broad, robust, its base and apex strongly divided, v-shaped. Penis capsule with inner arm well developed, outer arm short; penis broadening toward apex (Figure 9G); apex partially membranous, asymmetrical, with big, strongly curved hook (Figure 9H,I).

Female not studied.

**Distribution.** Republic of South Africa: Western Cape.

**Figure 10:**
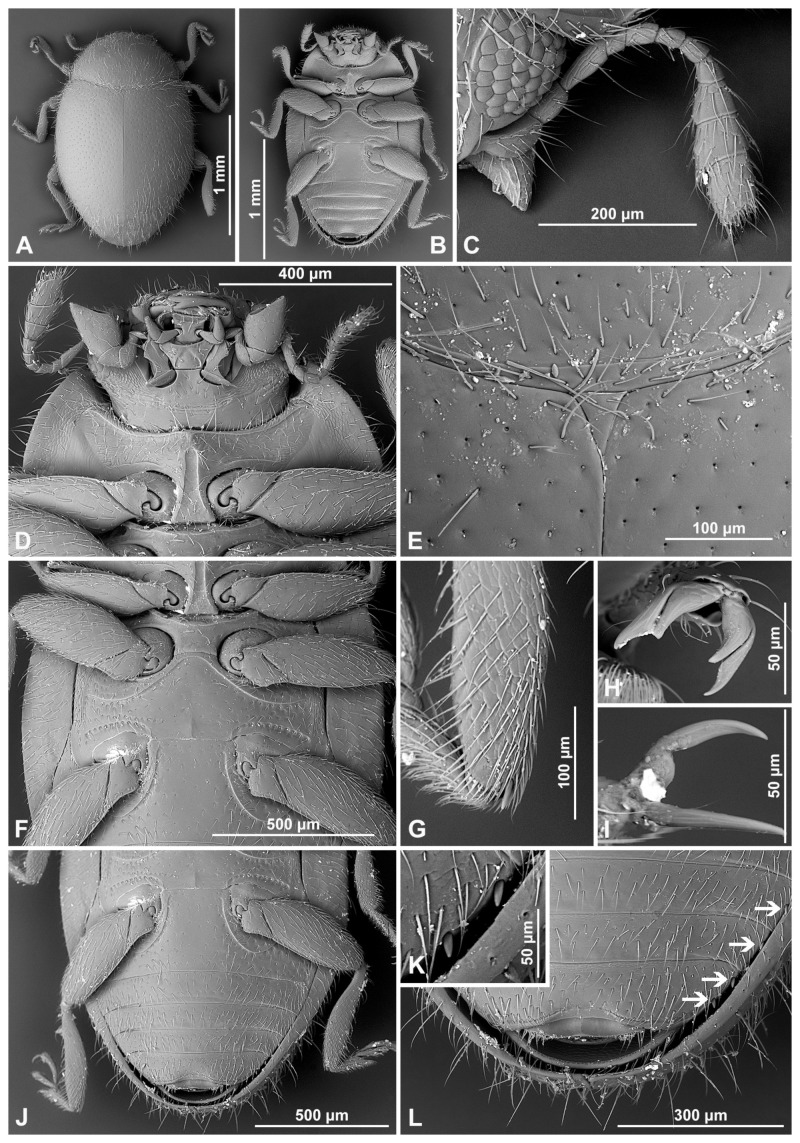
*Epipleuria natalensis* Fürsch, male (NHM). (**A**) Dorsal; (**B**) ventral; (**C**) antenna; (**D**) head and prothorax, ventral; (**E**) scutellar shield; (**F**) meso- and metathorax, ventral; (**G**) tibial spur on hind leg; (**H**) fore tarsal claws; (**I**) hind tarsal claws; (**J**) abdomen; (**K**) abdominal spines; (**L**) distal part of abdomen (arrows indicate abdominal spines).

**Figure 11:**
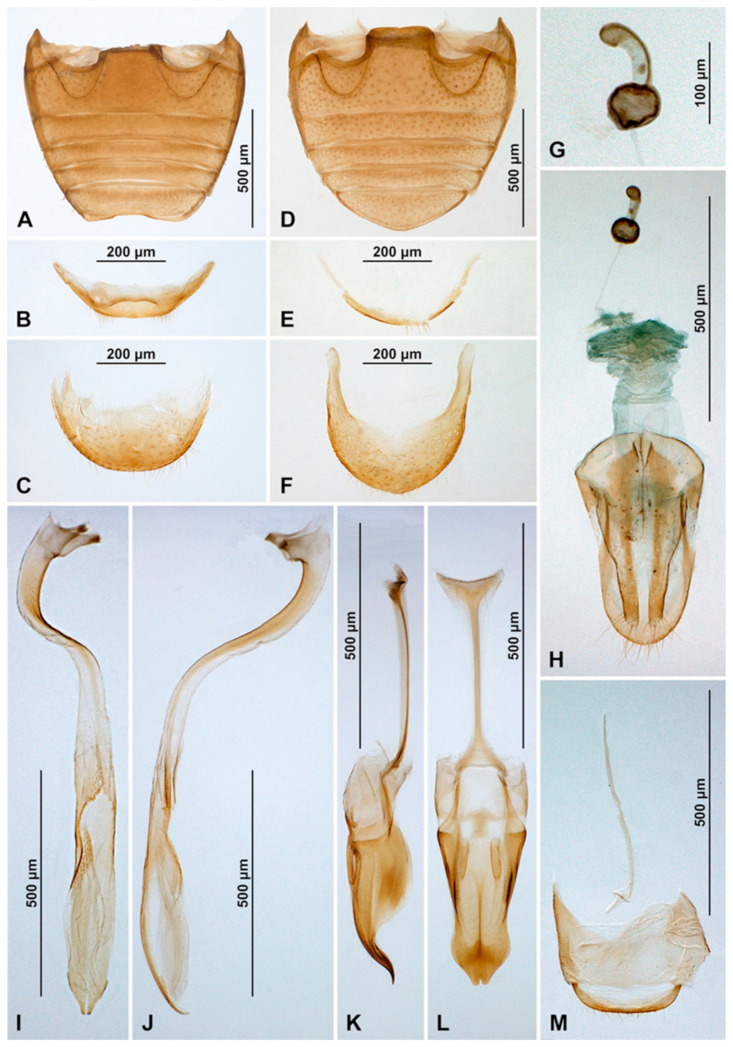
*Epipleuria natalensis* Fürsch (NHM). (**A**) Male abdomen; (**B**) male sternite VIII; (**C**) male tergite VIII; (**D**) female abdomen; (**E**) female sternite VIII; (**F**) female tergite VIII; (**G**) spermatheca; (**H**) female genitalia; (**I**) penis, inner; (**J**) penis, lateral; (**K**) tegmen, lateral; (**L**) tegmen, inner; (**M**) male segment IX.

***Epipleuria natalensis* Fürsch, 2001** (Figure 2J–K, Figure 10A–L and Figure 11A–M).
*Epipleuria natalensis* Fürsch, 2001: 21.

**Material examined.** “2159/Brit. Mus. 1922-431/ Estcourt, Natal. G.A.K. Marshall, 23.X.1892” (one male; NHM); “2158/ Brit. Mus. 1922-431/ Estcourt, Natal. G.A.K. Marshall, 23.X.1892” (one male; NHM); “2221/ Brit. Mus. 1922-431/ Estcourt, Natal. G.A.K. Marshall, 25.X.1892” (one male; NHM); “1181/ Brit. Mus. 1922-431/ Estcourt, Natal. G.A.K. Marshall, 25.VIII.1892” (one female; NHM).

**Redescription.** TL = 1.8–2.0 mm; EW = 1.1–1.2 mm; TL/EW = 1.6; PL/PW = 0.6; EL/EW = 1.2. Body elongate oval (Figure 2J and Figure 10A), moderately convex (Figure 2K). Dorsum covered with long, pale pubescence and sparse, erect bristles along lateral margin. Whole body uniformly brown.

Head. Interocular distance about 0.6× head width across eyes (Figure 2L). Antenna (Figure 10C) about equal as head capsule width; scape swollen, about 1.8 times longer than pedicel; antennomere 3 elongate, about 2.6 times longer than antennomere 4; antennomere 4 0.7× as long as antennomere 5; antennomeres 6 and 7 subquadrate; antennomere 8 slightly transverse; antennal club elongate, composed of three antennomeres, with two subterminal antennomeres transverse, asymmetrical, terminal antennomere elongate, about 1.6× as long as penultimate antennomere, its apex truncate. Maxilla with cardo transverse, distinctly reaching outside of mouth cavity. Labium with mentum transverse, anterior edge emarginate; labial palps separated by a distance of about equal to width of palpiger.

Prothorax. Anterior margin of pronotum with fine bordering line; lateral and posterior margins with entire border. Prothoracic hypomeron sparsely hairy, smooth, without groove or concavity (Figure 10D). Prosternum in front of coxa about 0.7× as long as coxal longitudinal diameter at the same position; anterior pronotal margin slightly produced medially, with bordering line incomplete medially; notosternal suture distinct; prosternal process narrow, about 0.6× as wide as corresponding procoxal width, with truncate apex, its surface with carinae converging, Y-shaped; procoxal cavity transverse, with bordering line incomplete, broadly separated from coxal cavity.

Pterothorax. Mesoventral process in middle about 0.7× as wide as corresponding coxal diameter; meso-metaventral articulation with suture visible; its junction straight (Figure 10F). Scutellar small but distinct, triangular, elongate (Figure 10E). Elytra with sides rounded; lateral margins narrow, visible from above only in basal half; humeral calli indistinct; elytral epipleuron in basal half about 5.3× as wide as corresponding metaepisternum, obsolete in apical half, not foveate (Figure 10B). Metaventrite with discrimen incomplete; metaventral postcoxal lines joined at middle forming arcuate line, complete and slightly recurved.

Legs. Trochanters angulately produced; tibiae broadened; tibial apices of mid and hind legs with single spur in both sexes (Figure 10G). In male fore and mid-tarsal claws bifid (Figure 10H), hind tarsal claws with subquadrate basal tooth (Figure 10I); in female claws in all legs with subquadrate basal tooth.

Abdomen with six ventrites in male (Figure 10J and Figure 11A) and five ventrites in female (Figure 11D); abdominal postcoxal lines separate medially, complete, rounded, posteriorly reaching about 0.6 times of length of ventrite 1; ventrite 1 below coxae about 1.7 times longer than ventrite 2; ventrites 3 and 4 equal in length, about 0.8 times of length of ventrite 2; ventrite 5 about 1.2 times longer than ventrite 4 in male and about 1.6 in female. Posterior margin of ventrite 5 in male distinctly emarginate medially (Figure 10L and Figure 11A), in female arcuate (Figure 11D). Spines on margins of ventrites 3–5 in male distinct (Figure 10K,L). In male tergite VIII rounded apically (Figure 11C), sternite VIII truncate with distinct median concavity (Figure 11B); in female tergite and sternite VIII rounded apically (Figure 11E,F).

Male terminalia and genitalia. Sternite IX (Figure 11M) with apodeme narrow, rod-like, base of apodeme widened, partially membranous; tergite X narrow, transverse. Tegmen in inner view (Figure 11L) with penis guide wide, symmetrical, its lateral margins sinuate, tip obtuse, slightly divided; penis guide in lateral view (Figure 11K) wide, its inner margin deeply emarginate, tip curved, pointed. Parameres narrow and short, about 0.3× as long as penis guide, its apices covered with long setae; tegminal strut broad, robust, its base widened and apex broadened, v-shaped. Penis capsule with outer arm well developed, inner arm reduced; penis broadening toward apex, covered by small spines; apex partially membranous, asymmetrical, slightly curved in lateral view (Figure 11J) and divided in inner view (Figure 11I).

Female genitalia (Figure 11H). Proctiger elongate with hind edge rounded, covered with long hairs. Coxites elongate, abruptly narrowed in the middle of the length; apical margins with row of sparse setae; styli distinct, bearing long setae. Sperm duct uniform in diameter, long; infundibulum absent; spermatheca (Figure 11G) with bulbous base and vermiform, curved apex, without clear nodulus and ramus, spermathecal accessory gland reduced.

Distribution. Republic of South Africa: KwaZulu-Natal.

**Figure 12:**
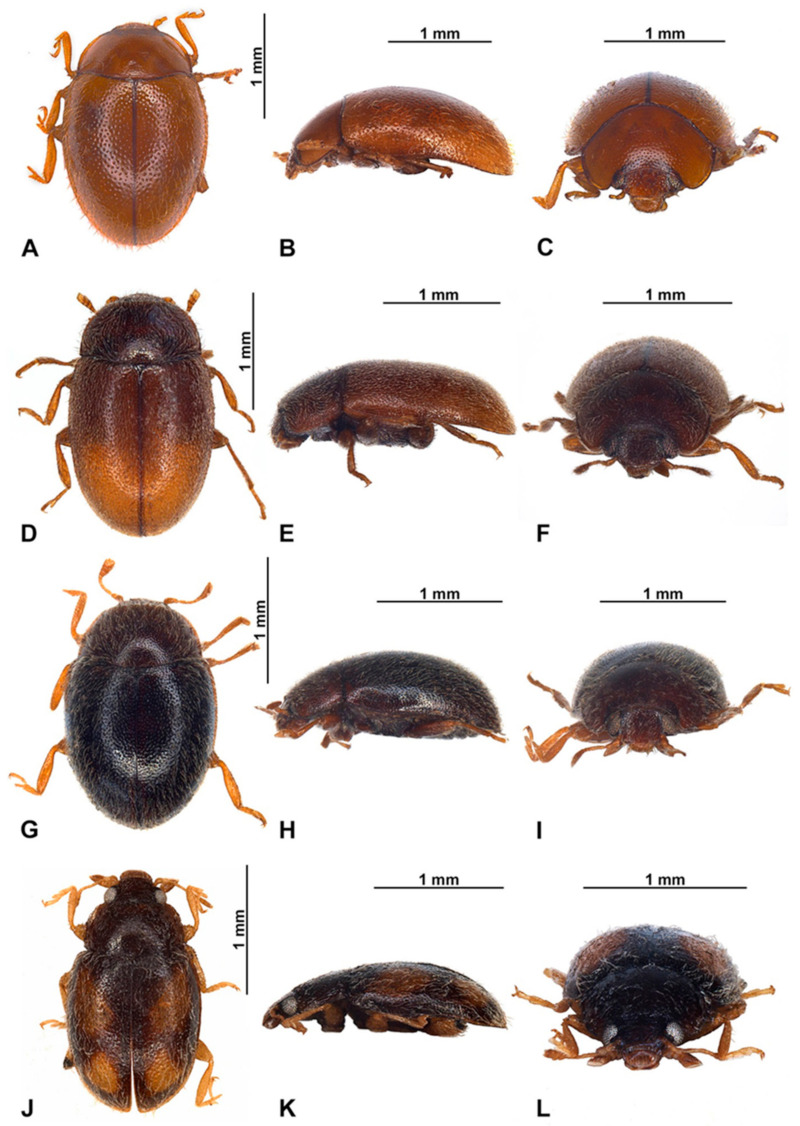
Habitus of *Epipleuria* Fürsch and *Pseudoepipleuria* gen. nov. (**A**–**C**) *Epipleuria tsitsikamma* sp. nov., holotype (DNMNH); (**A**) dorsal; (**B**) lateral; (**C**) frontal; (**D**–**F**) *Pseudoepipleuria endroedyi* (Fürsch) (MIZ); (**D**) dorsal; (**E**) lateral; (**F**) frontal; (**G**–**I**) *Pseudoepipleuria mahnerti* (Fürsch) (NHM); (**G**) dorsal; (**H**) lateral; (**I**) frontal; (**J**–**L**) *Pseudoepipleuria stillata* (Fürsch) (ZSM); (**J**) dorsal; (**K**) lateral; (**L**) frontal.

**Figure 13:**
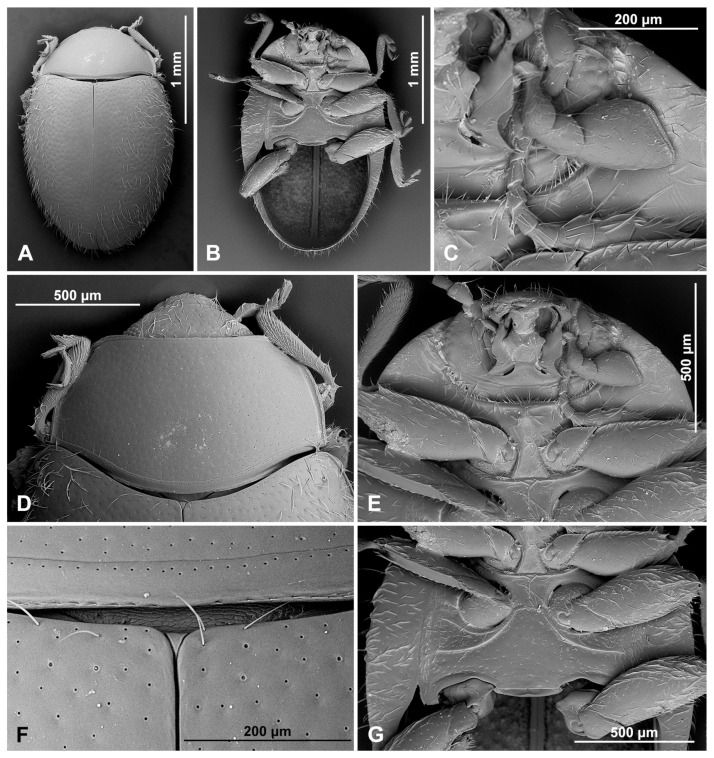
*Epipleuria tsitsikamma* sp. nov., male (DNMNH). (**A**) Dorsal; (**B**) ventral; (**C**) antenna; (**D**) pronotum; (**E**) head and prothorax, ventral; (**F**) scutellar shield; (**G**) meso- and metathorax, ventral.

**Figure 14:**
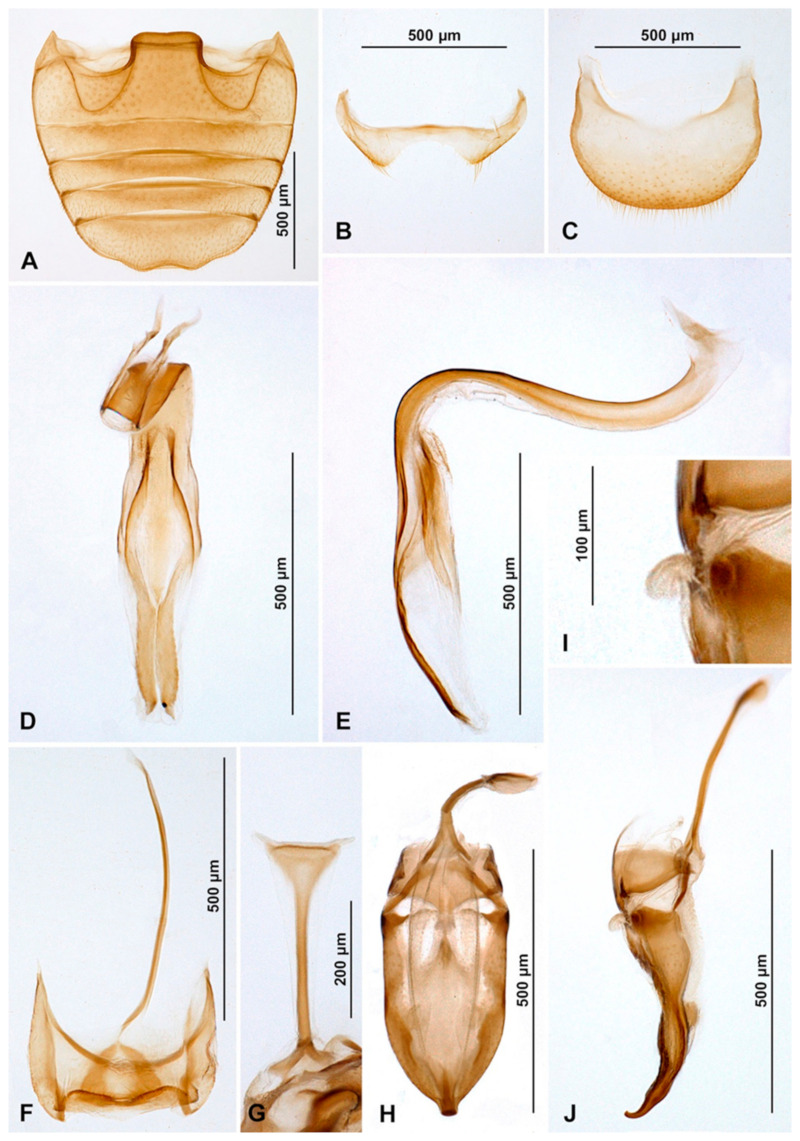
*Epipleuria tsitsikamma* sp. nov., male (DNMNH). (**A**) Abdomen; (**B**) sternite VIII; (**C**) tergite VIII; (**D**) penis, inner; (**E**) penis, lateral; (**F**) segment IX; (**E**) tegmen, inner; (**F**) tegmen, lateral; (**G**) tegminal strut; (**H**) tegmen, inner; (**I**) parameres; (**J**) tegmen, lateral.

***Epipleuria tsitsikamma* sp. nov.** (Figure 12A–C, Figure 13A–G and Figure 14A–J).
ZooBank. urn:lsid:zoobank.org:act:FA13A614-34B3-4FFA-B5CE-41AB6CE5CC79

**Material examined.** HOLOTYPE: male “RSA/ 24B W Cape 10–30 m -33.9678S/ 23.5604E Tsitsikamma N.P. Nature’s Valley, Grootkloof trail. km 0–2.5 beating 23.11.2013 leg. R. Ruta” (DNMNH).

**Etymology.** The name of this species refers to Tsitsikamma National Park, the locality where the holotype was collected.

**Diagnosis.** *Epipleuria tsitsikamma* sp. nov. is externally very similar to other species of the genus *Epipleuria*, but it can be distinguished from them via the following set of characteristics: scutellar shield very small but visible, discrimen indistinct, tibial apices of mid and hind legs without spurs, posterior margin of male ventrite 5 sinuate, and sternite VIII deeply emargined. The most reliable diagnostic character is provided by the genital structure (the parameres are strongly reduced to small lobes, while in other species, the parameres are at most shortened and never reduced to small lobes).

**Description.** TL = 2.2 mm; EW = 1.4 mm; TL/EW = 1.6; PL/PW = 0.5; EL/EW = 1.1. Body elongate oval (Figure 12A and Figure 13A), moderately convex (Figure 12B). Dorsum covered with long, yellowish pubescence and sparse, erect bristles along lateral margin. Whole body light brown, except coxae and femora darker.

Head. Interocular distance about 0.6× head width across eyes (Figure 12C). Antenna (Figure 13C) about equal as head capsule width; scape swollen, about 1.8 times longer than pedicel; antennomere 3 elongate, about 1.8 times longer than antennomere 4; antennomere 4 as long as antennomere 5; antennomere 6 subquadrate and antennomere 7 about 1.5× as long as wide; antennomere 8 subquadrate; antennal club elongate, composed of three antennomeres, with two subterminal antennomeres asymmetrical, terminal antennomere elongate, about 1.8× as long as penultimate antennomere, its apex truncate. Maxilla with cardo transverse, slightly reaching outside of mouth cavity. Labium with mentum transverse, anterior edge emargined; prementum subquadrate; labial palps separated by a distance of about equal to width of palpiger.

Prothorax. Anterior margin of pronotum with fine bordering line; lateral and posterior margins with entire border (Figure 13D). Prothoracic hypomeron sparsely hairy, smooth (Figure 13E). Prosternum in front of coxa about 0.6× as long as coxal longitudinal diameter at the same position; anterior pronotal margin prominent, with bordering line incomplete medially; notosternal suture distinct; prosternal process narrow, about 0.4× as wide as corresponding procoxal diameter, with truncate apex, its surface with carinae converging, Y-shaped; procoxal cavity transverse, with bordering line incomplete, broadly separated from coxal cavity.

Pterothorax. Mesoventral process in middle about 0.7× as wide as corresponding coxal diameter; meso-metaventral articulation with suture visible; its junction straight (Figure 13G). Scutellar shield very small but visible (Figure 13F). Elytra with sides rounded; lateral margins distinct, visible from above along whole margin; elytral epipleuron in basal half about 5.0× as wide as corresponding metaepisternum, obsolete in apical half, not foveate (Figure 13B). Metaventrite with discrimen indistinct; metaventral postcoxal lines joined at middle forming straight line, straight laterally, complete.

Legs. Trochanters simple; tibiae simple; tibial apices of mid and hind legs without spurs. Fore and mid-tarsal claws bifid.

Abdomen with five ventrites (Figure 14A); abdominal postcoxal lines separate medially, complete, rounded, posteriorly reaching about 0.7 times of length of ventrite 1; ventrite 1 below coxae about 1.4 times longer than ventrite 2; ventrites 2–4 equal in length; ventrite 5 about 1.4 times longer than ventrite 4. Posterior margin of ventrite 5 distinctly sinuate. Spines on margins of ventrites 3–5 absent. Tergite VIII rounded apically (Figure 14C); sternite VIII deeply emargined (Figure 14B).

Male terminalia and genitalia. Sternite IX (Figure 14F) with apodeme narrow, rod-like, base of apodeme strongly widened, partially membranous; tergite X transverse. Tegmen in inner view (Figure 14H) with penis guide wide, symmetrical, its lateral margins parallel and tip truncate, lateral sides of penis guide strongly upturned inwards; penis guide in lateral view (Figure 14J) partially membranous, tapering into apex, its inner margin sinuate, tip recurved and pointed. Parameres strongly reduced to small lobes (Figure 14I); tegminal strut broad, its base deeply divided and apex broadened, v-shaped (Figure 14G). Penis capsule with outer arm developed, inner arm reduced; penis broadening toward apex; its apex partially membranous in lateral view (Figure 14E), in inner view symmetrical, divided into two long lobes (Figure 14D).

Female not studied.

**Distribution.** Republic of South Africa: Western Cape.

**Genus *Pseudoepipleuria* gen. nov.**
Type species: *Epipleuria mahnerti* Fürsch, 2001—present designation.
ZooBank. urn:lsid:zoobank.org:act:F798353F-EEEE-404E-9ADC-E20F219DEC00

**Etymology.** The generic name is derived from the Latin pseudo (false) and *Epipleuria,* indicating the similar appearance of the new genus to *Epipleuria*. Gender: feminine.

**Diagnosis.** *Pseudoepipleuria* resembles the genus *Epipleuria* most by having the following characteristics: prosternal carinae converging anteriorly, Y-shaped, elytra fused along elytral suture, hind wings absent and tegmen with tegminal strut broad, robust, with divided and v-shaped apex. However, it can be distinguished from *Epipleuria* with the following characteristics: elytra covered with double elytral punctures (single in *Epipleuria*), large scutellar shield (in *Epipleuria* very small, sometimes indistinct); epipleura narrow, less than 3.0× as wide as corresponding metaepisternum (in *Epipleuria* more 4.0× as wide as corresponding metaepisternum), epipleura with bordering line along inner margin (bordering line absent in *Epipleuria*), tibiae with one or two apical spurs (in *Epipleuria* with single spur or without spurs), posterior margin of ventrite 5 in males rounded (variable in shape in *Epipleuria*), tegmen with penis guide simple and unmodified parameres (penis guide variable in shape, parameres strongly narrowed, sometimes shortened or reduced), penis simple, rod-like with apex simple, unmodified (penis robust, broadening toward apex, apex usually covered with spines or hooks) and spermatheca vermiform, C-shaped (spermatheca divided into bulbous base and vermiform, curved apex in *Epipleuria*). From the African members of *Rhyzobius,* it can be separated via the following characteristics: elytra covered with double elytral punctures (single in *Rhyzobius*), elytra fused along elytral suture (elytra not fused in *Rhyzobius*), hind wings absent (hind wings present in *Rhyzobius*) and tegminal strut broad, robust with divided and v-shaped apex (tegminal strut simple in *Rhyzobius*).

**Figure 15:**
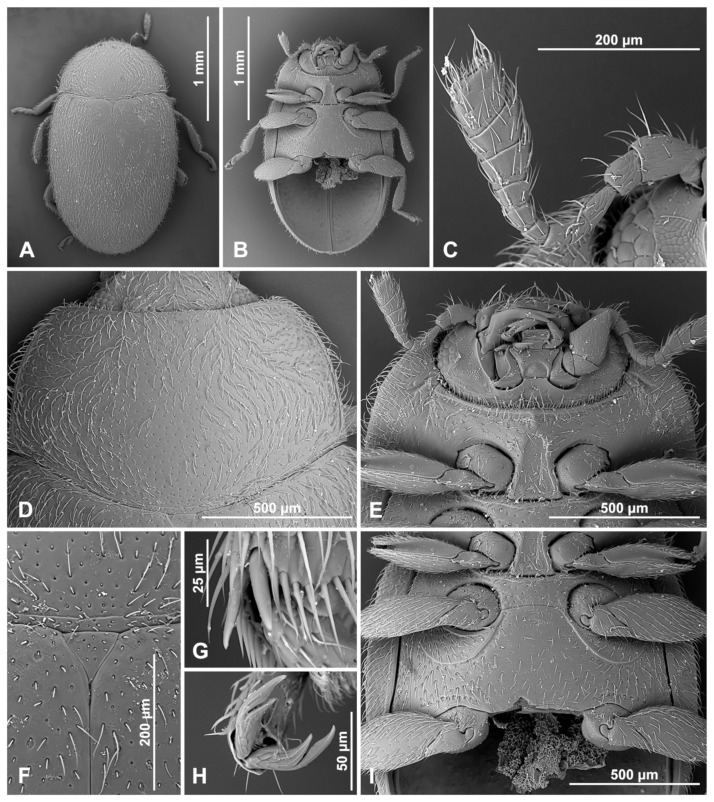
*Pseudoepipleuria endroedyi* (Fürsch), male (MIZ). (**A**) Dorsal; (**B**) ventral; (**C**) antenna; (**D**) pronotum; (**E**) head and prothorax, ventral; (**F**) scutellar shield; (**G**) tibial spurs on hind leg; (**H**) fore tarsal claws; (**I**) meso- and metathorax, ventral.

**Description.** Body elongate oval (Figure 12D,G,J), flattened to moderately convex (Figure 12E,H,K). Dorsum covered with uniform, moderately long pubescence with sparse, erect bristles along lateral margin. Elytra with double-sized punctures (Figure 15F); punctures on pronotum more dense than on elytra. Body unicolored brown, sometimes with pale maculae on elytra; antennae, mouthparts, legs and abdominal ventrites often paler, yellowish brown. Wingless.

Head partially covered by pronotum (Figure 12F,I,L); ventral antennal grooves indistinct. Eyes coarsely facetted; interfacetal setae present, sparse and short; eye canthus absent; interocular distance about 0.6–0.7× head width across eyes. Antenna about 0.8–1.0× as long as head capsule width, composed of 11 antennomeres; scape swollen, about 2 times longer than pedicel; pedicel barrel-shaped, distinctly narrower than scape; antennomere 3 elongate; antennal club composed of three antennomeres, with two subterminal antennomeres asymmetrical. Anterior clypeal margin straight, without lateral lobes. Labrum entirely exposed, transverse or rounded, truncate at apex. Maxilla with cardo transverse; maxillary stipes deeply foveate to accommodate maxillary palp in repose, basistipes with lateral carina distinctly sinuate; lacinia elongate, covered with moderately long setae; galea narrow, C-shaped, densely setose apically; maxillary palp composed of 4 palpomeres, palpomere 2 distinctly longer than palpomere 3; terminal palpomere subparallel, weakly expanded apically. Submentum short, subrectangular, its anterior margin straight. Labium with mentum transverse or subquadrate, anterior edge truncate or emargined; ventral surface with horseshoe-shaped median impression; prementum transverse or subquadrate; labial palps separated by distance of about 1.5x width of palpiger; palp composed of 3 palpomeres, terminal palpomere as long and as broad as penultimate, apically pointed.

Prothorax. Pronotum with anterior corners obtuse, not swollen with regular border; anterior margin with fine bordering carina; lateral and posterior margins with entire border (Figure 15D). Prothoracic hypomeron densely hairy, usually with oblique groove extending to about half of its width (Figure 15D). Prosternum in front of coxa about 0.6–0.8× as long as coxal longitudinal diameter at same position; anterior pronotal margin not produced medially, with bordering line incomplete medially; notosternal suture distinct or indistinct, straight; prosternal process about 0.6–0.7× as wide as corresponding coxal width, with truncate apex, its surface with carinae converging, Y-shaped; procoxal cavity weakly oval, without lateral slit, with or without bordering line.

Pterothorax. Mesoventrite transverse, its anterior edge emarginate medially with complete raised border (Figure 15I). Mesoventral process in middle about 0.8–1.2× as wide as corresponding coxal diameter; meso-metaventral articulation with suture visible; its junction straight. Scutellar shield moderately large, triangular (Figure 15F). Elytra fused along elytral suture (Figure 15A). Elytra with sides rounded or almost parallel-sided; lateral margins narrow; humeral calli indistinct; elytral epipleuron in basal half less than 3.0× as wide as corresponding metaepisternum, not foveate, inner margin with bordering line. Metaventrite with discrimen incomplete; metaventral postcoxal lines joined or separated medially, complete and recurved.

Legs. Trochanters simple or angulately produced; tibiae simple or somewhat broadened apically; tibial apices of mid and hind legs with single or double spurs. Tarsi pseudotrimerous, in males, fore and mid-tarsal claws bifid, hind tarsal claws with subquadrate basal tooth; in females, tarsal claws in all legs with subquadrate basal tooth.

Abdomen. Abdomen with five or six ventrites; abdominal postcoxal lines separate medially, complete, rounded, deep. Posterior margin of ventrite 5 in both sexes rounded. In males, tergite VIII rounded apically, sternite VIII slightly emarginate; in females, tergite and sternite VIII rounded apically.

Male terminalia and genitalia. Sternite IX with apodeme narrow, rod-like, widened at base, partially membranous, without additional sclerites; tergite X short, transverse, rounded apically. Tegmen with penis guide simple, symmetrical, wide at base and tapering toward apex. Parameres simple, not modified, its apices covered with long setae; tegminal strut robust, its base broadened and apex divided, v-shaped. Penis capsule with outer and inner arms well developed, inner arm narrow; penis narrow, rod-like, not broadening toward apex; apex not modified, not covered with hooks or spines.

Female genitalia. Sperm duct uniform in diameter; infundibulum absent; spermatheca C-shaped, vermiform, without clear nodulus and ramus, spermathecal accessory gland distinctly separated from sperm duct. Coxites elongate, subtriangular; apical margins with one row of sparse setae; styli distinct, bearing long setae.

**Distribution.** Republic of South Africa, Kenya.

Current composition of the genus (three species).

[Species examined in this study in bold, *—indicates the type species.]

*Pseudoepipleuria endroedyi* (Fürsch, 2001) comb. nov.; *Pseudoepipleuria mahnerti* (Fürsch, 2001) comb. nov. *; *Pseudoepipleuria stillata* (Fürsch, 1992) comb. nov.

**Figure 16:**
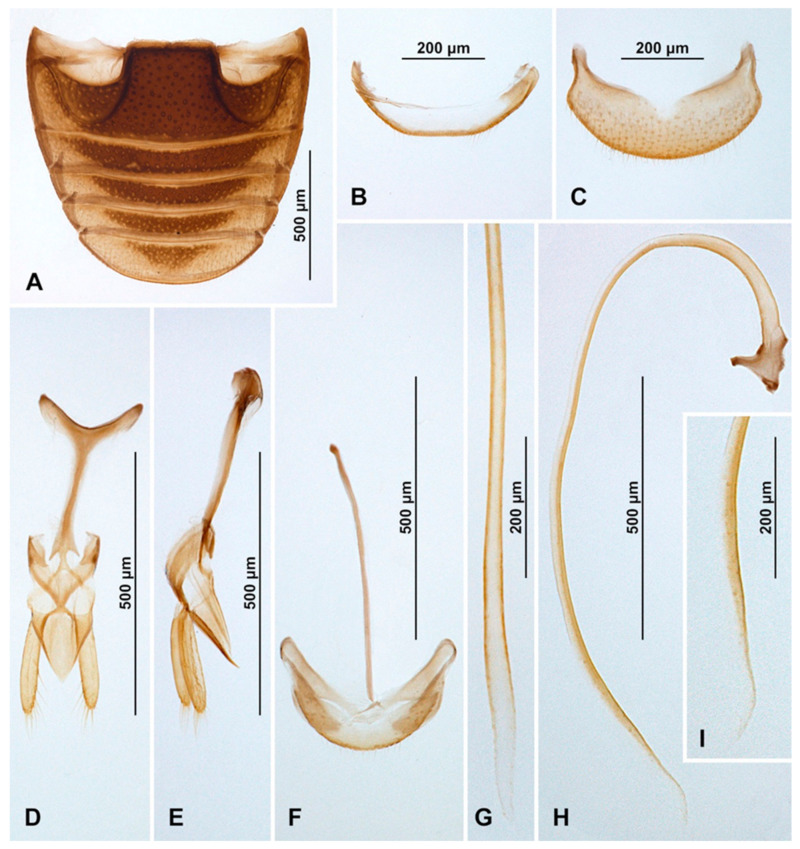
*Pseudoepipleuria endroedyi* (Fürsch), male (MIZ). (**A**) Abdomen; (**B**) sternite VIII; (**C**) tergite VIII; (**D**) tegmen, inner; (**E**) tegmen, lateral; (**F**) segment IX; (**G**) penis tip, inner; (**H**) penis, lateral; (**I**) penis tip, lateral.

***Pseudoepipleuria endroedyi* (Fürsch, 2001) comb. nov.** (Figure 12D–F, Figure 15A–I and Figure 16A–I).
*Epipleuria endroedyi* Fürsch, 2001: 8.

**Material examined.** “S.Afr., Namaqualand, Island Point, 4 km S, 30.56S – 17.38E/ 25.8.1979, E-Y: 1595 groundtraps, 63 days leg. Endrödy-Younga/ groundtraps meat bait/ Paratypoid *Epipleuria endroedyi* Fürsch 1986 [red card]/ MIZ PAN WARSZAWA 2/ 2019 499 [on reverse]” (one male; MIZ).

**Redescription.** TL = 2.2 mm; EW = 1.3 mm; TL/EW = 1.4; PL/PW = 0.5; EL/EW = 1.2. Body elongate oval (Figure 12D and Figure 15A), weakly convex (Figure 12E). Dorsum covered with uniform, moderately long, yellowish pubescence with sparse, erect bristles along lateral margin. Elytra with double-sized punctures, bigger punctures arranged into irregular rows. Body unicolored brown, with only antennae, mouthparts, tibiae, tarsi and lateral sides of abdominal ventrites paler, yellowish brown.

Head. Interocular distance about 0.6× head width across eyes (Figure 12F). Antenna (Figure 15C) about 0.8× as long as head capsule width; scape swollen, about 2 times longer than pedicel; antennomere 3 elongate, about 2 times longer than antennomere 4; antennomere 4 as long as wide and about 0.8× as long as antennomere 5; antennomeres 6 and 7 subquadrate; antennomere 8 0.8× as long as wide; antennal club composed of three antennomeres, with two subterminal antennomeres asymmetrical, transverse, terminal antennomere subquadrate, about 1.5× as long as penultimate antennomere, its apex truncate. Maxilla with cardo transverse, slightly reaching outside of mouth cavity. Labium with mentum weakly transverse, anterior edge truncate; labial palps separated by a distance of about 1.5× width of palpiger.

Prothorax. Anterior margin of pronotum with fine bordering carina; lateral and posterior margins with entire border (Figure 15D). Prothoracic hypomeron densely hairy, with oblique groove extending to about half of its width (Figure 15E). Prosternum in front of coxa about 0.8× as long as coxal longitudinal diameter at the same position; anterior pronotal margin not produced medially, with bordering line incomplete medially; notosternal suture hardly visible, straight; prosternal process about 0.7× as wide as corresponding procoxal width, with truncate apex, its surface with carinae converging, Y-shaped; procoxal cavity weakly oval, without bordering line.

Pterothorax. Mesoventral process (Figure 15I) in middle about 1.2× as wide as corresponding coxal diameter; meso-metaventral articulation with suture visible; its junction straight. Scutellar shield large, triangular, as long as wide, its surface punctate and setose (Figure 15F). Elytra with sides almost parallel-sided; lateral margins narrow, but visible from above; elytral epipleuron in basal half about 2.0× as wide as corresponding metaepisternum, not foveate, inner margin with border line fading before base of elytron (Figure 15B). Metaventral postcoxal lines separate medially, complete and recurved.

Legs. Trochanters simple; tibiae somewhat broadened apically; tibial apices of mid and hind legs with double spurs (Figure 15G). Fore and mid-tarsal claws bifid (Figure 15H), hind tarsal claws with subquadrate basal tooth.

Abdomen with five ventrites (Figure 16A); abdominal postcoxal lines separate medially, complete, rounded, deep, posteriorly reaching about 0.7 times of length of ventrite 1; ventrite 1 below coxae about 1.3 times longer than ventrite 2; ventrites 3 and 4 subequal in length; ventrite 5 longer than ventrite 4, its posterior margin rounded; tergite VIII rounded (Figure 16C) and sternite VIII truncate apically (Figure 16B).

Male terminalia and genitalia. Sternite IX (Figure 16F) with apodeme narrow, rod-like, its base divided, partially membranous; tergite X short, transverse, rounded apically. Tegmen in inner view (Figure 16D) with penis guide symmetrical, wide, its tip pointed; penis guide in lateral view (Figure 16E) simple, tapering into tip, tip obliquely truncate. Parameres simple, about 1.3× as long as penis guide, its apices covered with long setae; tegminal strut broad, robust, its base broadened and apex divided, v-shaped. Penis capsule with outer and inner arms well developed, inner arm narrow; penis narrow, rod-like, not broadening toward apex (Figure 16H); apex not modified, symmetrical and partially membranous in inner view (Figure 16G), its tip slightly curved in lateral view (Figure 16I).

**Distribution.** Republic of South Africa: Northern Cape.

**Remarks.** This species was originally described by Fürsch [9] as *Epipleuria endroedyi*. Since then, it has not been the subject of any studies. A detailed examination of the specimen deposited in MIZ revealed a number of characteristics distinguishing it from *Epipleuria* (double-sized punctures; scutellar shield distinct; epipleura narrow with border line, double tibial spurs, unmodified penis guide and parameres, penis rod-like with apex simple and spermatheca vermiform, C-shaped), and we propose to transfer it to the newly established genus *Pseudoepipleuria*.

**Figure 17:**
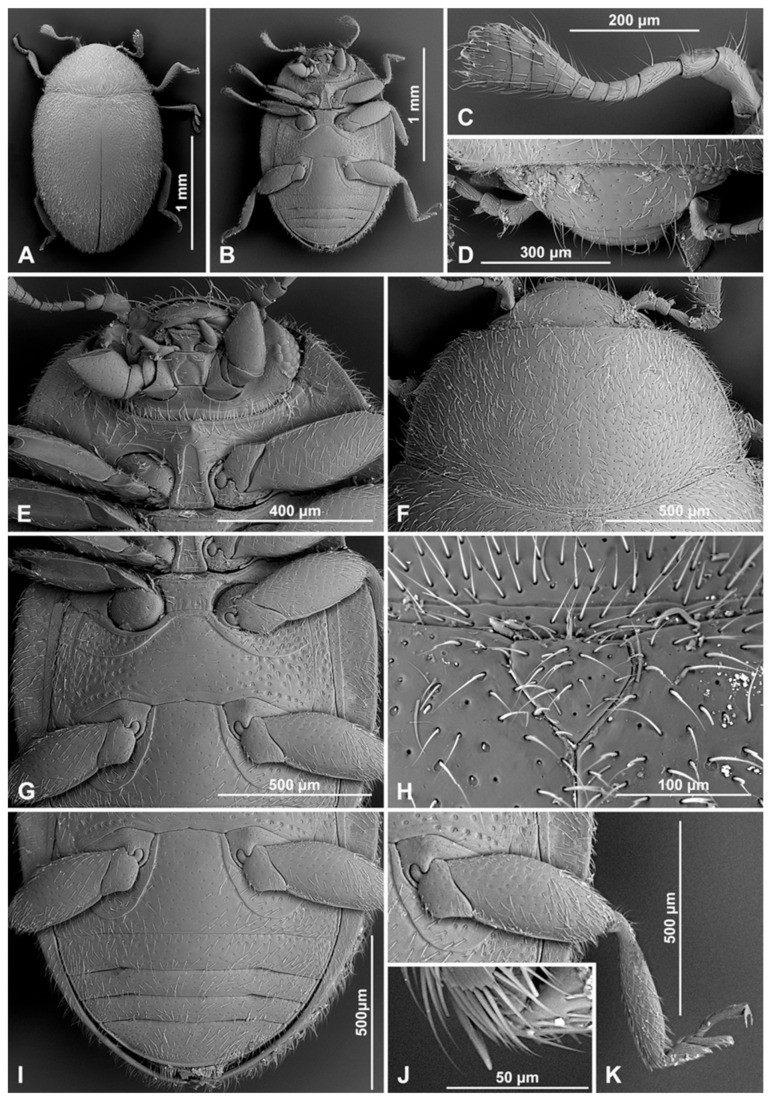
*Pseudoepipleuria mahnerti* (Fürsch), male (NHM). (**A**) Dorsal; (**B**) ventral; (**C**) antenna; (**D**) head, frontal; (**E**) head and prothorax, ventral; (**F**) pronotum; (**G**) meso- and metathorax, ventral; (**H**) scutellar shield; (**I**) abdomen; (**J**) tibial spur on hind leg; (**K**) hind leg.

**Figure 18:**
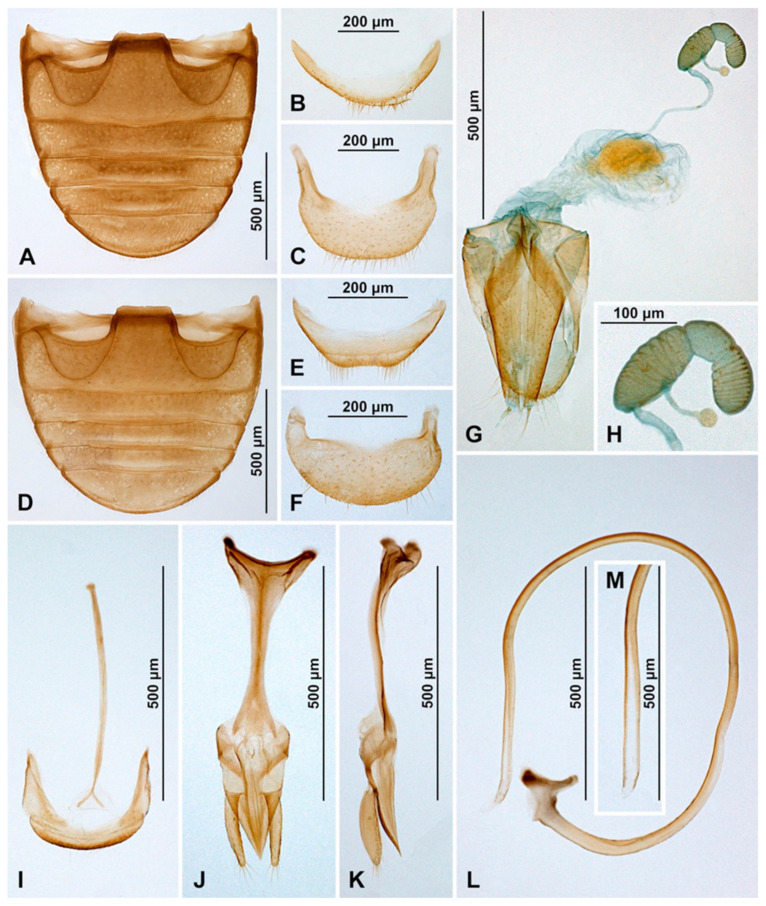
*Pseudoepipleuria mahnerti* (Fürsch) (NHM). (**A**) Female abdomen; (**B**) female sternite VIII; (**C**) female tergite VIII; (**D**) male abdomen; (**E**) male sternite VIII; (**F**) male tergite VIII; (**G**) female genitalia; (**H**) spermatheca; (**I**) male segment IX; (**J**) tegmen, inner; (**K**) tegmen, lateral; (**L**) penis, lateral; (**M**) penis tip, inner.

***Pseudoepipleuria mahnerti* (Fürsch, 2001) comb. nov.** (Figure 12G–I, Figure 17A–K and Figure 18A–M).
*Epipleuria mahnerti* Fürsch, 2001: 18.

**Material examined.** “Turner Kinangop March,-3Q/ Pres. by Comm Inst Ent B.M. 1981–315 [on reverse]” (one male, one female; NHM).

**Redescription.** TL = 1.8–2.1 mm; EW = 1.2–1.4 mm; TL/EW = 1.5; PL/PW = 0.5; EL/EW = 1.0. Body elongate oval (Figure 12G and Figure 17A), moderately convex (Figure 12H). Dorsum covered with long, pale pubescence and sparse, erect bristles along lateral margin. Body predominantly dark brown, almost black; antennae, mouthparts, legs except coxae, hypomera, epipleura and last abdominal ventrite paler, yellowish brown.

Head. Interocular distance about 0.7× head width across eyes (Figure 12I and Figure 17D). Antenna (Figure 17C) about 0.8× as long as head capsule width; scape swollen, about 1.5 times longer than pedicel; antennomere 3 elongate, about 2.5 times longer than antennomere 4; antennomere 4 0.7× as long as antennomere 5; antennomeres 6 and 7 as long as wide; antennomere 8 slightly transverse; antennal club short, compact, composed of three antennomeres, with two subterminal antennomeres distinctly transverse, asymmetrical, terminal antennomere subquadrate, about 1.5× as long as penultimate antennomere, its apex truncate. Maxilla with cardo transverse, not reaching outside of mouth cavity. Labium with mentum subquadrate, slightly expanded apically, anterior edge straight; labial palps separated by a distance of about 1.4× width of palpiger.

Prothorax. Anterior margin of pronotum with fine bordering line; lateral and posterior margins with entire border (Figure 17F). Prothoracic hypomeron densely hairy, with oblique groove extending to about half of its width (Figure 17E). Prosternum in front of coxa about 0.7× as long as coxal longitudinal diameter at the same position; anterior pronotal margin not produced medially, with bordering line incomplete medially; notosternal suture hardly visible; prosternal process narrow, about 0.6× as wide as corresponding procoxal width, with truncate apex, its surface with carinae converging, Y-shaped; procoxal cavity transverse, with bordering line incomplete, broadly separated from coxal cavity.

Pterothorax. Mesoventral process in middle about 0.8× as wide as corresponding coxal diameter; meso-metaventral articulation with suture visible; its junction straight (Figure 17G). Scutellar shield large, pentagonal, as long as wide, its surface punctate and setose (Figure 17H). Elytra with sides rounded; lateral margins narrow, visible from above only in basal half; elytral epipleuron in basal half about 2.6× as wide as corresponding metaepisternum, not foveate, inner margin with border line fading before base of elytra (Figure 17B). Metaventrite with discrimen incomplete; metaventral postcoxal lines joined at middle forming straight line, complete and recurved.

Legs. Trochanters somewhat angulately produced; tibiae simple (Figure 17K); tibial apices of mid and hind legs with single spur in both sexes (Figure 17J). In males, fore and mid-tarsal claws bifid, hind tarsal claws with subquadrate basal tooth; in females, claws in all legs with subquadrate basal tooth.

Abdomen. Abdomen with five ventrites in both sexes (Figure 17I and Figure 18A,D); abdominal postcoxal lines separate medially, complete, rounded, posteriorly reaching about 0.7 times of length of ventrite 1; ventrite 1 below coxae about 1.4 times longer than ventrite 2; ventrites 3 and 4 equal in length, about 0.8 times of length of ventrite 2; ventrite 5 about 1.3 times longer than ventrite 4. Posterior margin of ventrite 5 in both sexes rounded. In males, tergite VIII rounded apically (Figure 18F), sternite VIII slightly emarginate (Figure 18E); in female tergite (Figure 18C) and sternite VIII (Figure 18B) rounded apically.

Male terminalia and genitalia. Sternite IX with apodeme narrow, rod-like, base of apodeme widened, partially membranous; tergite X narrow, transverse (Figure 18I). Tegmen in inner view (Figure 18J) with penis guide wide, symmetrical, tapering into tip, tip pointed; penis guide in lateral view (Figure 18K) simple, its inner margin straight, tip not obliquely truncate, pointed. Parameres simple, slightly widened in lateral view, as long as penis guide, its apices covered with long setae; tegminal strut broad, robust, its base widened and apex divided, v-shaped. Penis capsule with outer and inner and arm well developed, inner arm narrow; penis narrow, rod-like, not broadening toward apex; apex not modified, symmetrical and partially membranous in inner view (Figure 18M), its tip slightly curved in lateral view (Figure 18L).

Female genitalia (Figure 18G). Proctiger elongate with hind edge rounded, covered with long hairs. Coxites subtriangular in shape, elongate; apical margins covered with sparse setae; styli distinct, bearing long setae. Sperm duct uniform in diameter, long; infundibulum absent; spermatheca (Figure 18H) C-shaped, vermiform, without bulbous base and clear nodulus and ramus, spermathecal accessory gland distinctly separated from sperm duct.

**Distribution.** Kenya: Kinangop.

**Remarks.** This species, since its description by Fürsch [9], has never been studied. Fürsch [9] mentioned that it is the only species of *Epipleuria* found in rotting wood. A detailed examination of this species shows that its morphology and genital structures better correspond with the newly established genus *Pseudoepipleuria* (see Diagnosis), and we propose to transfer it to this genus.

**Figure 19:**
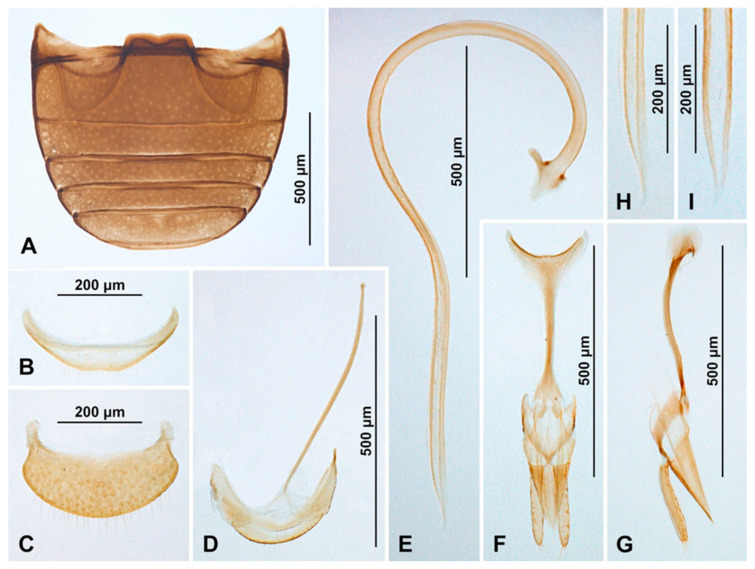
*Pseudoepipleuria stillata* (Fürsch), male (A. Ślipiński collection). (**A**) Abdomen; (**B**) sternite VIII; (**C**) tergite VIII; (**D**) segment IX; (**E**) penis, lateral; (**F**) tegmen, inner; (**G**) tegmen, lateral; (**H**) penis tip, lateral; (**I**) penis tip, inner.

***Pseudoepipleuria stillata* (Fürsch, 1992) comb. nov.** (Figure 12J–L and Figure 19A–I).
*Rhyzobius stillatus* Fürsch, 1992: 73.—Fürsch 2001: 6; Tomaszewska 2010: 48; Szawaryn & Tomaszewska 2020: 1455–1458.

**Material examined.** PARATYPE (Figure 12J–L): male “2505/ S. Afr., SW Cape Verlorevlei farm 32.19 S – 18.22 E/ 28.8. 1981; E-Y: 1857 shorewashing leg. Endrödy-Younga/ Paratypoid Rhyzobius stillatus Fürsch 1986 [red rectangle]/ Coll. Fürsch ZSM 2016/ SNSB-Zoologische Staatssammlung München Coleoptera ID # ZSM-COL-00594” (ZSM). Other material: “S. Africa Feb. 1927 R.E. Turner Brit. Mus.” (two males; A. Ślipiński collection).

**Emended description of abdomen and genitalia.** Abdomen with five ventrites (Figure 19A); abdominal postcoxal lines separate medially, complete, rounded, posteriorly reaching about 0.8 times of length of ventrite 1; ventrite 1 below coxae about 1.5 times longer than ventrite 2; ventrites 3 and 4 equal in length, about 0.8 times of length of ventrite 2; ventrite 5 about 1.3 times longer than ventrite 4. Posterior margin of ventrite 5 rounded. In males, tergite VIII rounded apically (Figure 19C), sternite VIII slightly emarginate (Figure 19B).

Male terminalia and genitalia. Sternite IX with apodeme narrow, rod-like, base of apodeme widened, partially membranous; tergite X narrow, transverse (Figure 19D). Tegmen in inner view (Figure 19F) with penis guide wide, symmetrical, tapering into tip, tip pointed; penis guide in lateral view (Figure 19G) simple, its inner margin straight, tip pointed. Parameres simple, as long as penis guide, its apices covered with long setae; tegminal strut broad, robust, its base widened and apex divided, v-shaped. Penis capsule with outer and inner and arm well developed, inner arm narrow; penis narrow, rod-like, not broadening toward apex (Figure 19E); apex not modified, symmetrical and partially membranous in inner view (Figure 19I), its tip slightly pointed in lateral view (Figure 19H).

**Remarks.** Fürsch, in the original description [8], noticed that this species is clearly different from the other species of *Rhyzobius*. In his subsequent publication, devoted to the genus *Epipleuria* [9], he discussed its systematic position and compared *R. stillatus* with both *Epipleuria* and *Rhyzobius*. Fürsch mentioned that *R*. *stillatus* cannot be easily assigned to any of the two genera. On the one hand, its body shape, distinct scutellar shield, markings on elytra and shape of spermatheca resembles the other species of *Rhyzobius*, but on the other hand, it looks like *Epipleuria* by having elytra fused together along the elytral suture, with a v-shaped tegminal strut and a lack of hind wings. Finally, Fürsch [9] left it in the genus *Rhyzobius*. Subsequently, this species was re-described in detail by Tomaszewska [2]. From other African species of *Rhyzobius,* it differs by having elytra covered with double elytral punctures, elytra fused along the elytral suture, a lack of hind wings and a tegminal strut with a divided apex that is v-shaped. On the other hand, it differs from *Epipleuria* by having the following characteristics: large scutellar shield, epipleura narrow with border line, double tibial spurs, unmodified penis guide and parameres, penis rod-like with apex simple and spermatheca vermiform, C-shaped. Thus, we propose the transfer of this species to the newly described genus *Pseudoepipleuria*.

## 4. Discussion

The Coccidulini fauna of the African realm is not very rich but has quite a convoluted history and, for a long time, remained poorly studied, partially due to the scarcity of collections. The majority of Coccidulini species are known from the Republic of South Africa, and a single species is known from Kenya. A single species of *Rhyzobius*, *R. c-pallidum,* is also known from Madagascar; however, its phylogenetic position is unclear.

In this study, we re-examined several species of *Epipleuria,* including the type species *Rhizobiellus epipleuralis* Pope. Based on microCT scans of the holotype of *E. epipleuralis* and an examination of other specimens from various collections and localities, we re-described the genus, which can be defined by the following characteristics: single-sized elytral punctures; scutellar shield very small or indistinct; lack of hind wings; elytra fused along elytral suture; epipleura strongly widened (over 4.0× as wide as corresponding metaepisternum); epipleura without border line; tibial apices with single spur or without spurs; tegminal strut broad, robust with divided and v-shaped apex; spermatheca divided into bulbous base and vermiform, curved apex. Based on this set of characteristics, it is well separated from the African species of *Rhyzobius*, which have the following characteristics: elytra with single-sized puncture; scutellar shield distinct; hind wings present; elytra not fused along elytral suture; epipleura narrow, less than 3.0× as wide as corresponding metaepisternum; tegminal strut narrow, unmodified and spermatheca vermiform, without bulbous base. Moreover, in contrast to species of *Rhyzobius*, where male genitalia are rather uniform, male genitalia in *Epipleuria* are very distinctive at the species level, including the shape of the tegmen, parameres and the tip of penis, which usually has variously shaped spines and hooks.

During our study, we noticed a group of species that clearly differ from both *Epipleuria* and *Rhyzobius* by having the following characteristics: double-sized elytral punctures; scutellar shield large; lack of hind wings; elytra fused along elytral suture; epipleura narrow (less than 3.0× as wide as corresponding metaepisternum); epipleura with border line; tibial apices with single or double spurs; tegminal strut broad, robust with divided and v-shaped apex; spermatheca vermiform, C-shaped. With this mix of characteristics, *Pseudoepipleuria* gen. nov. stands between *Epipleuria* and *Rhyzobius*; however, it is clearly different from both.

Although 24 species of *Epipleuria* are known so far, their biology is poorly known. Fürsch [9] mentioned that all species are terricolous and xerophilous, and they live on the ground in contrast to *Rhyzobius* species, which live on various types of vegetation. Moreover, *Epipleuria* specimens were collected using ground traps filled with bananas, meat or excrements, while the specimens of *Rhyzobius* are most often collected by the beating of plants.

## Data Availability

All data is contained within the article.
